# Effects of immersion in a simulated natural environment on stress reduction and emotional arousal: A systematic review and meta-analysis

**DOI:** 10.3389/fpsyg.2022.1058177

**Published:** 2023-01-09

**Authors:** Hongyi Li, Yujun Ding, Bing Zhao, Yuhang Xu, Wei Wei

**Affiliations:** ^1^The College of Landscape Architecture, Nanjing Forestry University, Nanjing, China; ^2^The College of Environmental Science, Nanjing Xiaozhuang University, Nanjing, China

**Keywords:** immersion, natural environment, stress, emotion, restorative effect, virtual reality

## Abstract

**Background:**

Although the mental health benefits of exposure to simulated natural environments are well established by researchers from environmental psychology, landscape architecture, and public health, it is unclear whether and to what extent technological immersion affects these benefits.

**Methods:**

Systematical literature searches were conducted in May 2022 from six databases. The risk of bias was evaluated using the Cochrane’s Risk of Bias tool 2.0 and the Risk of Bias in Non-randomized Studies of Interventions tool. We performed a random-effects meta-regression to investigate the heterogeneity. The immersion levels of included studies were classified by projection devices and motion capture, and then subgroup analysis was conducted.

**Results:**

Twenty-six publications were included. Exposure to simulated nature was confirmed to be associated with increased positive affect 0.40 [95% confidence interval (CI): 0.22, 0.58], vigor 0.58 (95% CI: 0.30, 0.86), calmness 0.54 (95% CI: 0.17, 0.92) and decreased perceived stress −0.38 (95% CI: −0.71, −0.06), total mood disturbance −0.87 (95% CI: −1.17, −0.57), tension −0.70 (95% CI: −0.99, −0.41), fatigue −0.60 (95% CI: −0.91, −0.28), anxiety −0.72 (95% CI: −1.43, −0.02), depression −0.33 (95% CI: −0.52, −0.14), confusion −0.79 (95% CI: −1.19, −0.40), and anger −0.54 (95% CI: −0.76, −0.31). Gender, health status, study design, mean age, and single exposure duration were not significant when entered in a meta-regression. For positive affect, medium immersion was observed to produce a larger effect than low and high immersion. All included studies had a moderate to high risk of bias.

**Conclusion:**

Audio-visual exposure to simulated nature contributes to stress relief and emotional arousal. The immersion level explains the heterogeneity of positive affect triggered by simulated nature. Focusing on the technical features will open up new possibilities for combining actual and simulated nature’s mental health benefits.

## 1. Introduction

The effective use of the psychological health benefits of the natural environment has become a key component of Nature-Based Solutions (Bauduceau et al., 2015) for sustainable urbanization in the societal dimension (van den Bosch and Ode Sang, 2017). However, physical access to nature is sometimes restricted by various factors, resulting in urbanities being deprived of the opportunity to enjoy nature (Bratman et al., 2019). Evidence suggests that various dimensions of urban green space (UGS) exposure measures are likely to impact the biopsychosocial pathways linking UGS to human health and, thus, might induce significant disparities in health outcomes (Zhang J. et al., 2020; Zhang, 2023). Urban heat islands (Huang et al., 2016; Elliott et al., 2020), the busy lifestyle of urbanites (Oguz and Cakci, 2010), and smartphone addiction (Richardson et al., 2018; Gao et al., 2022) make even those who are expected to have access to ample UGS lack the time or willingness to visit.

The perceived or cultural accessibility aspects of Nature-Based Solutions are often overlooked compared to physical accessibility (van den Bosch and Ode Sang, 2017). However, they contribute prominently to enhancing aesthetic values and self-reported well-being (Ode Sang et al., 2016). Measures to strengthen people’s connection to nature are considered likely to be more effective than improving UGS availability (Lin et al., 2014). Studies on simulated nature are at the forefront of this field. A simulated nature experience has been shown to attract young people to digital media over outdoor recreation (Pergams and Zaradic, 2006) and to facilitate the protection of seniors from environmental hazards and barriers (Satariano et al., 2012; Wen et al., 2018). Crucially, human-nature connections can be enhanced by exposure to simulated natural environments, and people may be more inclined to visit their actual counterparts (Litleskare et al., 2020; Chan et al., 2021b).

Immersion refers to the way computer displays convey an immersive, extensive, surrounding, and vivid illusion of reality to human senses (Slater and Wilbur, 1997). It has been demonstrated that higher immersion leads to an increased sense of presence experienced by users in mediated environments (Baños et al., 2004), thereby magnifying user effects (e.g., the similarity between user responses to virtual stimuli and parallel responses to real-world counterparts; Cummings and Bailenson, 2015) and allowing real emotions to be activated (Parsons and Rizzo, 2008; Price et al., 2011). Thus, several researchers are banking on using VR as an alternative to inferior immersive projection devices (e.g., computer screens, television screens, etc), which have often been considered limiting factors in previous studies (Zabini et al., 2020; Jiang et al., 2021). It is empirically believed that immersive virtual environment technology will more fully exploit the psychological health benefits of simulated natural environments. However, this statement is based on a hypothesis lacking sufficient evidence to support it—that higher levels of immersion are associated with greater restorative potential *via* the natural environment mediating the experience. Direct evidence on the moderating effect of immersion on restorative effects is conflicting (de Kort et al., 2006; Kjellgren and Buhrkall, 2010). Two of them found no significant differences in skin conduct level, heart rate (HR), or perceived relaxation between the head-mounted display (HMD) and computer screen projecting the identical blue space video (Villani et al., 2007; Knaust et al., 2021). Viewing simulated green and blue spaces through an HMD may even lead to a more active rather than relaxed cortical brain (Reece et al., 2022). On the other side, Yeo et al. (2020) claimed that computer-generated immersive natural environments had a facilitative effect on positive affect. A study based on Attention Restoration Theory explained that high immersion promoted being away and perceived fascination (Karacan et al., 2021). Nevertheless, such direct evidence is limited in quantity and with insufficient statistical power due to the small sample sizes.

For psychological and physiological benefits, the validity of highly immersive nature simulations should be reexamined (Browning M. H. E. M. et al., 2020). A recent systematic review found inconsistencies in reducing negative affect by exposure to an immersive simulated nature, with no significant positive effect differences (Frost et al., 2022). Another suggested that accessing virtual environments with natural stimuli through HMD may effectively promote relaxation from stressful states in the general population (Riches et al., 2021). However, a brief qualitative synthesis of technological immersion has been included only in the broader reviews related to methodological choices and modalities (Browning M. H. E. M. et al., 2020; Nukarinen et al., 2022). In the absence of a more targeted review, the question of how much immersion in simulated nature is enough remains unanswered.

The objective of the present study was to quantify the effects of immersion level on the restorative effects of simulated nature, which are restricted to stress or emotional outcomes. The systematic review and meta-analysis allowed us to integrate a large number of available empirical researches with different immersion levels. Specifically, we focused on audio-visual experiments since, among the environmental stimuli that have the most significant impact on humans, visual and auditory stimuli are the two primary information sources people perceive from their surroundings (Preis et al., 2015). People living in high-density urban areas are more likely to experience a more diverse and complex audio-visual environment (Jahncke et al., 2015). Their restoration demands should be met both visually and auditorily. Audio-visual experiments are the most widespread evidence for multisensory modalities (Browning M. E. H. M. et al., 2020; Nukarinen et al., 2022), allowing for a review of this type of evidence feasible and realistic. Three hypotheses will be tested in the current study:

*Hypothesis 1*: Audio-visual exposure to simulated nature contributes to stress relief and emotional arousal.

*Hypothesis 2*: Immersion level can explain the variability in the simulated natural restorative effects across studies.

*Hypothesis 3*: Higher immersion in simulated natural environments is associated with more stress reduction and emotional arousal.

## 2. Materials and methods

The current review was conducted in compliance with the Preferred Reporting Items for Systematic Reviews and Meta-Analyses (PRISMA) guidelines (Moher et al., 2009). The PRISMA checklist is available in Supplementary Table S1. The protocol can be accessed on the PROSPERO, number CRD42022345184.

### 2.1. Search strategy

The electronic literature search covered six databases (PsycINFO, PubMed, Embase, Cochrane Library, Web of Science, Scopus). The search terms were derived from previous reviews on the natural environment and psychological well-being (Bowler et al., 2010; McMahan and Estes, 2015; Jo et al., 2019; Roberts et al., 2019; Browning M. H. E. M. et al., 2020; Yao et al., 2021; Frost et al., 2022). We performed pre-retrieval to optimize the search strategy by replacing the word “nature” with phrases that more accurately express the natural environment and adding keywords related to humans. The official search terms related to five topics, i.e., natural environment (e.g., “natural environment,” “natural space*,” “greenness” or “green space*”), simulation (e.g., “virtual real*,” “immersi*” or “simulation*”), psychological effects (e.g., “mental health,” “emotion*,” “stress” or “restorative*”), subject (e.g., “participant,” “human,” “m?n” or “wom?n”), and study design (e.g., “randomized controlled trial,” “non-randomized,” “intervention” or “exposure”). Neither publication year nor region restrictions were applied, but references only published or in press in English were included, without books, abstracts, or gray literature searches. The deadline for both the pre-retrieval and the official search was May 2020. See Supplementary Table S2 for the complete search strategies for each database.

### 2.2. Study selection

The bibliographic records from each database were downloaded and integrated into Endnote 20, where duplicates were removed. Two reviewers (HL and YD) screened each record separately for title and abstract and removed irrelevant articles, with a 98% agreement rate. The full text of all eligible articles was then evaluated. The consensus was achieved through discussion or referral to a third reviewer (BZ). The PICOS framework was used to guide the development of eligibility criteria.

Population: No restrictions on gender, age or health status.

Intervention: Simulated natural environment.

Comparator: Simulated non-natural environment.

Outcome: Stress reduction and emotional arousal.

Study: Experimental studies.

A simulated natural environment was broadly defined as an audio-visual projection of an existing or fictional setting dominated by vegetation and/or other abiotic natural features (Browning M. E. H. M. et al., 2020), excluding animals. It should be noted that the simple nature-build dichotomy is increasingly being questioned due to the difficulty of providing urban planning guidance and exaggerating the differences between environments (Van den Berg et al., 2014; Brancato et al., 2022). Here, we classify urban scenes dominated by natural elements as natural environments as well, aiming to revise and deepen our understanding of the essence of restorative environments.

Studies containing sensory stimuli other than audio-visual stimuli or evaluating imaginary environments (Korpela and Hartig, 1996; Korpela et al., 2008) would be excluded. The presentation of visual and auditory information should be synchronized in a time sequence and matched in content, as a real experience requires consistent sensory impressions (Sona et al., 2019). Users are limited in their tolerance for temporal or spatial incongruence between visual and auditory stimuli (Kim and Lee, 2022). Simulated biophilic indoor environment and blue space were not included. The study of additional physical exercise (Duncan et al., 2014; White et al., 2015; Yeh et al., 2017; Wooller et al., 2018; Alkahtani et al., 2019), driving behavior (Jiang et al., 2020), and psychological interventions, such as mindfulness programs (Choe et al., 2020), would also be beyond the scope of this study.

The main outcomes of this meta-analysis were related to psychological indicators of stress and emotion, which should be assessed by standardized quantitative measurement. Both self-reported outcome measures and psychophysiological parameters were permitted. We included only Experimental studies in the systematic review and meta-analysis. A control group of non-natural environments was required for eligible articles. Otherwise, it would be difficult to determine whether psychological outcomes have changed due to simulated nature or projection devices (Snell et al., 2018). Studies with pre- and post-tests or continuous monitoring were eligible. Consistency should be maintained between the experimental group and control group in terms of tasks performed, duration of exposure, and measurement.

### 2.3. Data extraction

A standardized, predesigned extraction form in Microsoft Excel 2016 was developed by the first author (HL) to extract the following information for each study meeting the inclusion criteria: reference information, subject, experimental design, group exposure, duration, devices, motion capture, outcome measures, and main conclusion. Another author (YX) verified the data extraction, and all disagreements were resolved through discussion, resulting in a consensus.

### 2.4. Criteria for immersion levels

Despite the lack of uniform criteria for defining immersion levels in simulations, projection devices and motion capture have been highlighted as two well-defined and identifiable technical features in empirical researches (Villani et al., 2007; Yeo et al., 2020; Karacan et al., 2021; Knaust et al., 2021; Reece et al., 2022) and reviews (Cummings and Bailenson, 2015; Miller and Bugnariu, 2016; Beck et al., 2019; Smith, 2019; Hatta et al., 2022) to differentiate the immersion level. Therefore, a criterion for evaluating overall immersion through projection devices and motion capture was established (Table 1). In a study, we first assessed the level of immersion according to the projection devices and motion capture used. Based on this, the overall immersion level was determined. If the study received a low immersion rating in both technical features, it was classified as a low immersion study. The study was classified as medium immersion in cases where at least one technical feature was categorized as medium immersion. Generally, a study is classified as overall high immersion only if both the projection device and motion capture are highly immersive.

**Table 1:** Criteria of immersion levels.

Immersion level	Projection devices	Motion capture	Overall immersion
Low	Non-surround projection devices without isolation from physical reality (e.g., computer screen, television screen, projector screen)	No motion capture; the user’s perception of self-location and possible actions is absent	Classified as low immersion in both projection devices and motion capture
Medium	CAVEs with limited isolation from physical reality	Head motion capture providing a free view to match head movement	One or both of the projection devices and motion capture are classified as medium immersion
High	HMDs capable of complete isolation from physical reality	Head and body motion capture. They provide a free field of view simultaneously allowing displacement through proprioceptive motion in the simulated space	Classified as high immersion in both projection devices and motion capture

CAVE, cave automatic virtual environment; HMD, head-mounted display.

### 2.5. Risk of bias and quality of evidence assessment

The Cochrane Risk of Bias (ROB) Tool 2.0 was used to access the included randomized controlled studies. Selection bias, performance bias, detection bias, attrition bias, and reporting bias were judged for each eligible study. Referring to the ROB 2.0 detailed guidance for the signaling questions and the suggested decision tree, the results of the five evaluation domains were given, and the overall bias was then ascertained (Sterne et al., 2019).

For the non-randomized controlled study, we chose the Risk of Bias in Non-Randomized Studies of Interventions (ROBINS-I) tool, which was recommended by the Cochrane Scientific Committee. Seven fields were evaluated: confounding bias, selection bias, classification bias, deviation bias, attrition bias, measurement bias, and reporting bias (Sterne et al., 2016). Two reviewers (HL and YX) independently evaluated all studies, and appraisals were discussed to reach a consensus.

The Recommendations for Assessment, Development, and Evaluation (GRADE) guideline (Guyatt et al., 2008) was used to assess the quality of evidence and strength of recommendation for the main outcomes. The quality of evidence is classified as high, moderate, low, or very low and the strength of recommendation is classified as strong and weak. Several domains were identified to assess the certainty of evidence. Study design, risk of bias, inconsistency, indirectness, imprecision, and publication bias are factors that reduce the certainty of evidence. In contrast, large effect sizes, plausible residual confounding, and dose–response gradients can enhance the certainty of the evidence.

### 2.6. Narrative summary and meta-analysis

After reviewing each article, a narrative summary was prepared to integrate the main features of all included studies. To avoid serious bias, only randomized studies were included in the meta-analysis. Studies should provide an effect size or sufficient information [e.g., mean and standard deviation (SD)] to calculate an effect size. GetData Graph Digitizer was used to extract the data in the form of a statistical graph. A request for complete information was emailed to the author if the data were not included in the article. The following rules were developed to ensure the comparability of the extracted data: pre-test and post-test data were extracted simultaneously; only the data at the two time points closest to the start and end of the exposure were extracted if individuals were measured multiple times during the experiment; some continuously measured psychophysiological outcomes were reported for multiple periods (Wang et al., 2016; Snell et al., 2018; Mostajeran et al., 2021; Chan et al., 2021a,b), where the closest pre-exposure period and exposure period were used; following the recommendations of the Cochrane Handbook, when no intervention group in common was present in a multi-arm study, the results of the independent pair-wise comparison were included. Otherwise, all intervention groups’ means and standard deviations would be combined to create a single pair-wise comparison (Higgins et al., 2019). A study involving a control group and multiple experimental groups projecting different natural scenes would typically apply to this method. However, the data are not combined if a group meets any of the exclusion criteria.

The change scores for each study’s experimental and control groups were computed. We used a random effects model since it is considered to be more conservative, and appropriate for cases of high heterogeneity (Deeks et al., 2019). For each outcome measure, the standardized mean difference (SMD) was applied as the summary statistic in the meta-analysis if the included studies used different measures. Otherwise, the mean difference (MD) was calculated. Because the sample size is generally small in this emerging field (Browning M. et al., 2020; Riches et al., 2021; Frost et al., 2022), a standardized Hedge’s *g* rather than Cohen’s *d* was chosen due to its less bias under such circumstances (Hedges, 1981). The empirical rule states that 0.2 represents a small effect, 0.5 represents a medium effect, and 0.8 represents a large effect (Cohen, 1988). Each eligible data item was entered into the RevMan 5.4 and presented as forest plots with 95% confidence intervals (CIs). The I^2^ statistic was used to test the heterogeneity; 30–60% is considered moderate heterogeneity, and > 60% represents substantial heterogeneity (Deeks et al., 2019). We used a leave-one-out sensitivity analysis to investigate the robustness of the pooled effect estimate.

Univariate meta-regression was used to investigate the sources of heterogeneity possibly arising. It applied to only outcome measures with more than 10 studies reported (Deeks et al., 2019). The six independent variables for the univariate meta-regression were gender, mean age, health status, single exposure duration, immersion level, and study design. We used a random-effects meta-regression model with restricted maximum likelihood. The Covariate for the subgroup analyses was pre-specified as the immersion level. Each subgroup was required to contain at least two studies. The potential publication bias was visually inspected by funnel plots for outcome measures with more than 10 studies reported. Their asymmetry was quantified by Egger’s test and Begg’s test. Subgroup analysis and meta-regression were completed using RevMan 5.4 and the “metareg” macro in STATA 16. All statistical tests were two-sided.

## 3. Results

**Figure 1:**
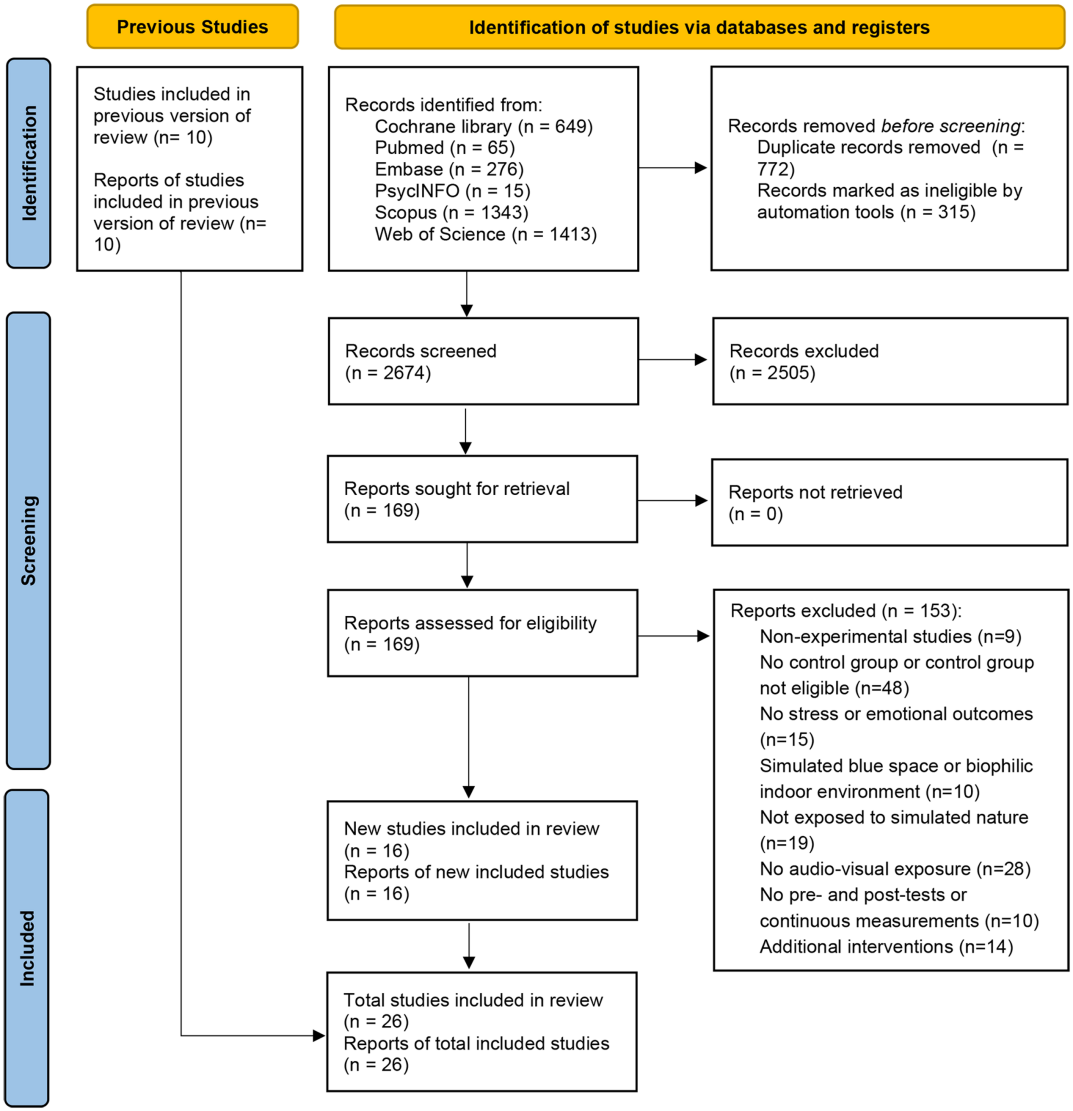
The PRISMA flow diagram version 2020.

The initial database search yielded a total of 3,761 records, of which 169 were eligible for full-text review. Ten additional records were retrieved from checking reference lists and review articles. After the independent assessment, 28 studies from 26 publications met the inclusion criteria. The data identification, screening, eligibility, and inclusion process are detailed in Figure 1. Refer to Supplementary Table S3 for a summary of the characteristics and outcomes of each study.

### 3.1. Publication years and locations

The article meeting the inclusion criteria first appeared in 2003 (van den Berg et al., 2003). Although no restrictions were imposed on the publication year, the number of publications after 2014 was 92.3% (*n* = 24). A steady increase was witnessed from 2020 onward. This suggests that simulated nature and psychological well-being has become a hot topic for researchers to explore after the coronavirus disease 2019 (COVID-19) outbreak. Twenty-eight studies came from 15 different countries and regions. The United States (*n* = 4) and the United Kingdom (*n* = 4) were the two countries with the highest number of publications. A cross-continental study convened subjects from both the United States and Hong Kong (Jiang et al., 2021).

### 3.2. Subjects

The sample sizes ranged from 20 (Chan et al., 2021b) to 269 (Bornioli et al., 2018), with a median of 69. Studies with larger sample sizes (n ≥ 200) were mainly conducted *via* online platforms (Bornioli et al., 2018; Brancato et al., 2022). More than half of the studies had subjects exclusively from universities or research institutions. Of these, 16 were conducted with undergraduate and graduate students, and three included a mix of students and university or institution employees (Annerstedt et al., 2013; Kinnafick and Thøgersen-Ntoumani, 2014; Bornioli et al., 2018). There was a skew toward youth in most studies, with 70.8% of the subjects being under 30 years old and only one study specifically aimed at seniors (mean age: 72.7; Chan et al., 2021b). Three studies included clinical populations who suffered from mild depression, stress, anxiety (Jo et al., 2022), depressive symptoms (Meuwese et al., 2021), or high exam anxiety (O’Meara et al., 2020).

### 3.3. Immersion levels and exposure durations

Twenty-five studies did not use motion capture (*n* = 15) or used only head motion capture (*n* = 10) during the subjects’ viewing of the simulated environment. Among these, 15 studies used non-surround projection devices. None of the studies employing online surveys declared the use of audio-visual materials adapted to Cave Automatic Virtual Environment (CAVE) or HMD (Bornioli et al., 2018; Zabini et al., 2020; Brancato et al., 2022). Of the remaining 10 studies, HMDs were the most frequently utilized surround projection devices (*n* = 9), with CAVE being used in only one study (Annerstedt et al., 2013). Three studies employed head and body motion capture to induce displacement in simulated spaces through proprioceptive movements (Chan et al., 2021a,b; Newman et al., 2022), all of which used HMDs. In summary, the studies defined low, medium, and high immersion levels as 15, 10, and 3, respectively.

The duration of individual scene exposure in all studies ranged from 1 min (Bornioli et al., 2018) to 40 min (Annerstedt et al., 2013), with 50% of studies less than 6 min 20 s. The most common individual scene exposure durations were 5 min (*n* = 5) and 15 min (*n* = 5). One study involved repeated exposures over 5 days (Zabini et al., 2020). For both randomized and non-randomized crossover designs, the cumulative exposure duration for all settings, including the control condition, ranged from a minimum of 6 min (Chan et al., 2021b) to a maximum of 48 min (Mostajeran et al., 2021). To minimize the carryover effects caused by the crossover design, subjects were generally asked to undergo an interval after the exposure session, usually after 1 week (*n* = 3) or within a week (*n* = 2). One study provided rest periods as needed by the subjects (Jo et al., 2022), and another did not specify how long the rest period was (Mostajeran et al., 2021).

### 3.4. Visual and auditory materials

Visually, forests were the most frequently used simulated nature type (*n* = 14), followed by parks (*n* = 6). Four studies set up multiple simulated natural environments (Van den Berg et al., 2014; Wang et al., 2016; Brancato et al., 2022). Some studies used the broad term “natural environment” or “natural landscape” without emphasizing specific types (Schutte et al., 2017; McMahan et al., 2018; Snell et al., 2018; Newman et al., 2022). Subjects in two studies viewed urban scenes dominated by natural elements, including vertical greenery attached to the exterior of a building (Chan et al., 2021a) and the tree-lined neighborhood (Brancato et al., 2022). Comparator descriptions varied and covered commercial areas (Kinnafick and Thøgersen-Ntoumani, 2014; Bornioli et al., 2018; Jo et al., 2022), downtown areas (Zabini et al., 2020; Chan et al., 2021a), urban streets and roadways (van den Berg et al., 2003, 2014; Pilotti et al., 2014; Wang et al., 2016; Schutte et al., 2017; Jiang et al., 2021). They differed in pedestrian volume, traffic conditions, and building density, but all included little or no natural elements. Five studies were conducted using a blank control, i.e., exposure to an environment without simulated visual and auditory input (Annerstedt et al., 2013; Sona et al., 2019) or a monochromatic visual background (Snell et al., 2018; Browning et al., 2019; Kimura et al., 2021).

A total of 17 studies used live recordings of audio and visual samples, while three studies used computer-generated environments with matching audio samples (Jiang et al., 2021; Chan et al., 2021a,b). They were typically downloaded and edited from online audio material repositories. Kimura et al. (2021) used videos posted online. Among the nature audio contents, birdsong (*n* = 11), running water (*n* = 6), and wind (*n* = 4) were the most common. Traffic noise (*n* = 9) and conversations (*n* = 5) represented iconic built environment sounds. Jiang et al. (2021) utilized a mixture of the same type of audio to simulate a complex acoustic environment in a real environment by superimposing various sound sources over one another, which may have led the subjects to feel that the simulated environment mirrored reality in some way.

### 3.5. Outcome measures

Of the 28 studies, 53.57% used only self-reported psychological indicators, and the remaining used at least one psychophysiological indicator. Subjects primarily assessed perceived emotion using the Positive and Negative Affect Scale (PANAS; *n* = 10) and the Profile of Mood States (POMS; *n* = 9), followed by the State–Trait Anxiety Inventory (STAI; *n* = 5). For perceived stress, two studies used single-item scales (van den Berg et al., 2003; Chan et al., 2021b). Nine studies evaluated the psychophysiological response. Heart rate variability (HRV; *n* = 9) is a widely used psychophysiological indicator. The next was electrodermal activity (EDA; *n* = 5) and HR (*n* = 4). In three studies, systolic blood pressure (SBP) and diastolic blood pressure (DBP) were used in combination. Salivary cortisol, salivary α-amylase activity, and T-wave amplitude were used one time each.

### 3.6. Risk of bias

**Figure 2:**
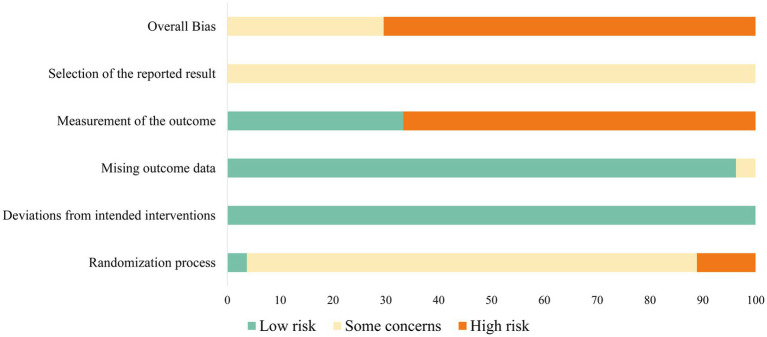
Cochrane risk of Bias 2.0 graph.

Twenty-seven randomized control studies were evaluated using the Cochrane ROB Tool 2.0 (Figure 2 and Supplementary Figure S1). Six studies used computer procedures to generate random numbers (McMahan et al., 2018; Snell et al., 2018; Jiang et al., 2021; Meuwese et al., 2021; Chan et al., 2021a), and the remaining articles stated only that the studies were randomized. Twenty-five studies did not provide information on whether the allocation sequence was concealed, one study was suspected of not achieving allocation sequence concealment given that the experimental recruiters developed an information sheet containing the subjects’ schedules (Bielinis et al., 2020). Another study declared the use of a procedure to schedule random numbers (Meuwese et al., 2021). However, no apparent baseline differences were found between intervention groups except in one study (O’Meara et al., 2020). Thus, most studies that were unclear in randomization methods and the concealment of allocation sequence still received a “some concern” rating in the randomization process domain. For the domain of deviations from intended interventions, the nature of the experiment was such that performing blinding on subjects was impossible. However, implementation of the intervention was successful for most participants, resulting in all studies being rated as low risk.

Two studies obtained data from all or almost all (≥95%) of the subjects (Meuwese et al., 2021; Jo et al., 2022). The common reasons for missing data were technical errors in data collection and subjects not completing the self-reported scales as needed, but missingness in the outcome was not related to the true value. Thus, almost all studies were rated as “low risk” in the domain of missing outcome data. However, a study with an elderly population was rated as “some concern” because the physical condition of the subjects may lead to excessive abnormal values in the HRV data (Chan et al., 2021b). A high ROB is broadly present in the measurement of the outcome. A significant proportion of studies relied only on subjects’ self-reported outcomes. The subjects themselves, as outcome assessors, had access to interventions with a high likelihood of guessing the experimental hypothesis and producing social desirability effects (Bowler et al., 2010), so up to 18 studies were considered high risk.

One non-randomized controlled study was evaluated using the ROBINS-I (Supplementary Table S4). A series of crucial confounding factors were identified, including the subject’s physical and psychological health status, nicotine, alcohol, and caffeine intake, auditory/visual interference in the laboratory, the subject’s familiarity with the equipment, and the presence of other elements in the scene that might affect psychological state (e.g., people, animals, conspicuous signs, etc). Mostajeran et al. (2021) noted that these elements are excluded. However, the subjects’ health status was unclear and therefore had a moderate risk of confounding bias. The study had low selection bias as subjects were not selected according to participant characteristics observed after the intervention began. Interventions were defined explicitly, and their classification was not influenced by knowledge or risk of the outcome; thus, the bias in the classification of interventions was considered low. The subjects in the study complied with the intervention. Finally, the study protocols for neither randomized nor non-randomized controlled studies were available, leaving us unable to judge whether the results were selectively reported.

### 3.7. Meta-analysis

#### 3.7.1. Effect size and heterogeneity

Two studies were not included in the meta-analysis due to missing data (McMahan et al., 2018) or not separately reporting stress and emotion outcomes (Jiang et al., 2021). At least one set of necessary statistical information was extracted from the remaining randomized studies, enabling meta-analyses of 22 commonly reported outcome measures. The estimates are pooled in Table 2. Statistically significant health denoting associations between the experimental and control groups were identified for self-reported psychological outcomes (positive affect, perceived stress, total mood disturbance, tension, fatigue, vigor, anxiety, depression, confusion, anger, and calmness). Exposure to simulated nature had a large significant positive effect on reducing total mood disturbance −0.87 (*p* < 0.00001), with a medium effect on increasing positive affect 0.40 (*p* < 0.0001) and recovering perceived stress −0.38 (*p* = 0.02 < 0.05). Meta-analyses of psychophysiological outcomes suggested that exposure to simulated nature may lead to a slight but not statistically significant reduction in physiological stress. Supplementary Table S5 specifies the main emotional and stress outcomes.

**Table 2:** Summary meta-analysis results table: the pooled effect sizes between simulated nature and non-nature exposure groups.

Outcome	Outcome test	Number of studies (participant)	Effect MD or SMD	95% CI	Heterogeneity I^2^	*p* value	Summary
Self-reported psychological outcomes							
Positive affect	PANAS, ZIPERS, single-item scale	11 (849)	0.40	(0.22, 0.58)	37%	<0.0001*	Improved
Negative affect	PANAS, ZIPERS, POMS	10 (848)	−0.09	(−0.23, 0.05)	0%	0.20	No effect
Perceived stress	single-item scale	2 (146)	−0.38	(−0.71, −0.06)	0%	0.02*	Improved
TMD	POMS	2 (188)	−0.87	(−1.17, −0.57)	0%	<0.00001*	Improved
Tension	POMS, AD ACL	8 (767)	−0.70	(−0.99, −0.41)	73%	<0.00001*	Improved
Fatigue	POMS	5 (380)	−0.60	(−0.91, −0.28)	55%	0.0002*	Improved
Vigor	POMS. SVS	5 (518)	0.58	(0.30, 0.86)	55%	<0.0001*	Improved
Anxiety	STAI	4 (444)	−0.72	(−1.43, −0.02)	91%	0.04*	Improved
Depression	POMS	5 (438)	−0.33	(−0.52, −0.14)	0%	0.0006*	Improved
Confusion	POMS	3 (248)	−0.79	(−1.19, −0.40)	54%	<0.0001*	Improved
Anger	POMS	4 (318)	−0.54	(−0.76, −0.31)	0%	<0.00001*	Improved
Happiness	VAS	2 (284)	0.26	(0.00, 0.51)	0%	0.05	No effect
Calmness	AD ACL, VAS, single-item scale	3 (378)	0.54	(0.17, 0.92)	63%	0.004*	Improved
Arousal	Nitsch’s Personal State Scale, single-item scale	3 (161)	−0.10	(−0.41, 0.21)	0%	0.54	No effect
Valence	UWIST-MACL, Nitsch’s Personal State Scale, single-item scale	3 (206)	0.40	(−0.67, 1.48)	92%	0.46	No effect
Psychophysiological outcomes							
SBP	/	3 (189)	**0.86**	(−3.88, 5.61)	54%	0.72	No effect
DBP	/	3 (189)	**−0.49**	(−2.71, 1.73)	0%	0.67	No effect
HR	/	4 (188)	−0.10	(−0.40, 0.20)	5%	0.08	No effect
LF power	HRV	2 (88)	0.22	(−0.20, 0.64)	0%	0.31	No effect
HF power	HRV	3 (148)	0.24	(−0.15, 0.63)	24%	0.22	No effect
LF/HF	HRV	2 (80)	−0.10	(−0.54, 0.34)	0%	0.66	No effect
RMSSD	HRV	2 (131)	0.11	(−0.23, 0.45)	0%	0.53	No effect

MD, mean difference; SMD, standardized mean difference; TMD, total mood disturbance; SBP, systolic blood pressure; DBP, diastolic blood pressure; HR, heart rate; LF, low frequency; HF, high frequency; RMSSD, root mean square on successive differences; PANAS, positive and negative affect schedule; ZIPERS, Zuckerman’s inventory of personal reactions scale; POMS, profile of mood states (including its short form and translated form); AD ACL, the activation-deactivation adjective check list; SVS, subjective vitality scale; STAI, state–trait anxiety inventory; VAS, visual analogue scale; UWIST-MACL, the University of Wales Institute of Science and Technology Mood Adjective Checklist; HRV, heart rate variability.

*p < 0.05. Bolded fonts indicate MD, non-bold fonts indicate SMD. Summary describes the effect of exposure to simulated nature on outcome measures.

Zero heterogeneity was reported for 11 of the analyses. Five reported moderate heterogeneity (30–60%), and six reported substantial heterogeneity (> 60%). Sensitivity analyses indicated that meta-analysis estimates were robust for more widely reported outcome measures such as positive affect (*n* = 11) and negative affect (*n* = 10), but not for some of them with fewer included studies (Supplementary Tables S6–S27).

#### 3.7.2. Meta-regression

Univariate meta-regression was only applicable to positive affect (*n* = 11). A significant variation in change scores was observed between high and medium immersions (*p* = 0.002 < 0.05, 95% CI: 0.23, 1.01) but not between high and low immersions (*p* = 0.852, 95% CI: −0.27, 0.33). There was insufficient evidence to support that gender (*p* = 0.131, 95% CI: −0.27, 2.11, R^2^ = 0%), health status (*p* = 0.407, 95% CI: −0.58, 0.24, R^2^ = 0%), study design (*p* = 0.743, 95% CI: −0.48, 0.34, R^2^ = 0%), mean age (*p* = 0.087, 95% CI: 0, 0.26, R^2^ = 15.91%) or single exposure duration (*p* = 0.192, 95% CI: 0, 0, R^2^ = 2.25%) moderate the effect of exposure to simulated nature on positive affect (Table 3).

**Table 3:** Summary meta-regression results table.

Moderator	Classification	Number of studies	*P* value	R^2^ (%)
Dichotomous and multi-category outcomes				
Health status	Nonclinical population	8	Ref	0
	Clinical population^1^	3	0.407	
Immersion level	High	3	Ref	100
	Medium	4	0.002*	
	Low	4	0.852	
Study design	Non-randomized	3	Ref	0
	Randomized	8	0.743	
Continuous outcomes				
Gender (male proportion)		11	0.131	0
Mean age		11	0.087	15.91
Single exposure duration		11	0.192	2.25

*p < 0.05.

^1^The clinical population refers to people suffering from depression and anxiety symptoms.

#### 3.7.3. Subgroup analysis

**Figure 3:**
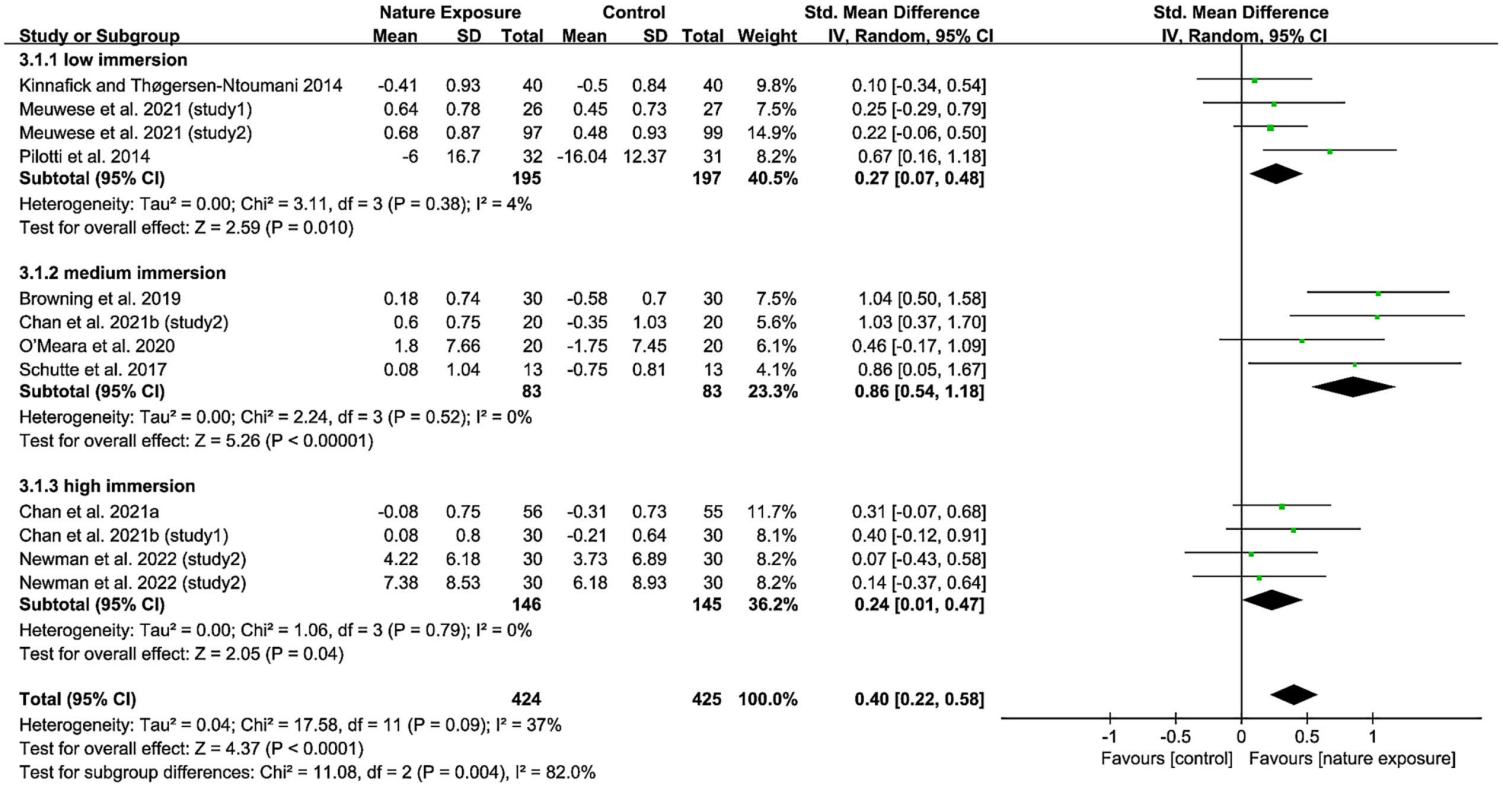
Subgroup analysis of the effects of simulated nature exposure on positive affect.

The subgroup analysis for positive affect supported the results of the meta-regression. The immersion level as a covariate led to a significant reduction in subgroup heterogeneity compared to the overall heterogeneity (low immersion: I^2^ = 4%, medium immersion: I^2^ = 0%, high immersion: I^2^ = 0%). Significant subgroup differences were also observed (I^2^ = 82%). We found that medium immersion produced a large effect 0.86 (95% CI: 0.54, 1.18), while low immersion 0.27 (95% CI: 0.07, 0.48) and high immersion 0.24 (95% CI: 0.01, 0.47) had a small effect (Figure 3). No subgroup differences were observed in the negative affect (Supplementary Figure S2).

**Figure 4:**
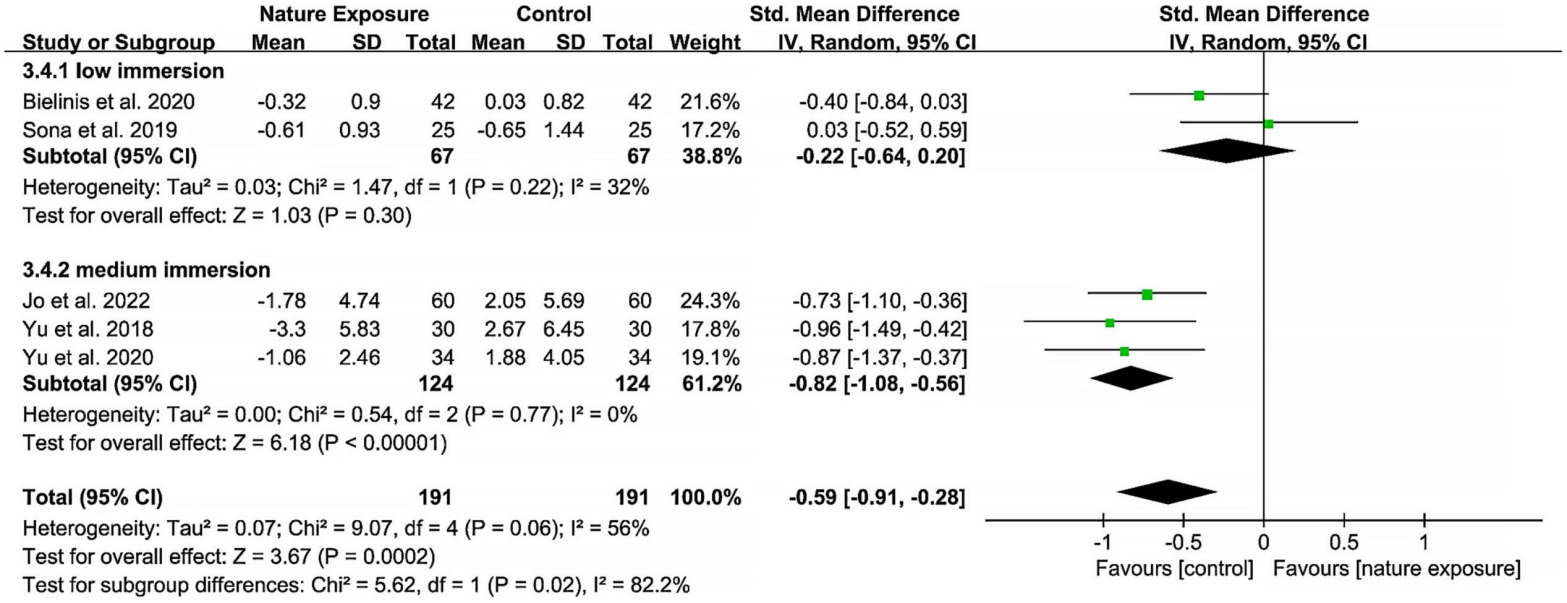
Subgroup analysis of the effects of simulated nature exposure on fatigue.

For the adverse event of fatigue, the pooled estimate was negative, indicating that exposure to simulated nature could inhibit the production of fatigue. The effect size of medium immersion −0.82 (95% CI: −1.08, −0.56) was lower than that of low immersion −0.22 (95% CI: −0.64, 0.20), suggesting a more pronounced inhibitory effect of the former (Figure 4). The outcome measures whose heterogeneity was not explained by the immersion level were tension, vigor, and calmness (Supplementary Figures S3–S5). A subgroup analysis for perceived or physiological stress was impossible due to the limited number of studies.

#### 3.7.4. Publication bias

Visual inspection of the funnel plot for both positive and negative affect indicated that they were approximately symmetric (Supplementary Figures S6, S7). For positive affect, Begg’s test (*p* = 0.011 < 0.05) suggested a significant publication bias, while Egger’s test (*p* = 0.455) did not detect a publication bias. Both Begg’s (*p* = 0.436) and Egger’s (*p* = 0.590) tests of funnel asymmetry were not significant in negative affect, suggesting that the effects of publication bias were negligible.

### 3.8. Quality of evidence

In line with GRADE guidelines, the randomized studies were firstly classified as high quality evidence. However, downgrades occurred in the domains of risk of bias and imprecision. Each study was at moderate or high risk of bias, leading to the overall risk of bias being considered serious or very serious. Second, for the remaining seven main outcome measures [negative affect, SBP, DBP, HR, LF (low frequency) power, HF (high frequency) power, LF/HF, RMSSD (Root Mean Square on Successive Differences)] other than positive affect and self-reported stress, the confidence intervals for their effect sizes crossed the clinical decision thresholds for both recommended and non-recommended interventions. This makes them considered to have very serious imprecision. Additionally, the overall sample size with only positive affect meets the criteria for optimal information content (OIS). In sum, the certainty of all the evidence is below moderate. The summary of findings table was presented in Supplementary Table S28.

## 4. Discussion

Increasingly, simulated nature is being offered as a distinctive “natural prescription” to improve the mental and physical health of individuals who are homebound or limited in their mobility (White et al., 2018). However, associating higher immersion with more significant restorative effects may be an empirical conjecture. A central finding of this review was that audio-visual exposure to simulated nature contributes to stress relief and emotional arousal. Immersion levels explained the heterogeneity in the pooled estimate for positive but not negative affect. Given the small sample sizes and the lack of low ROB studies, limited confidence must be maintained in the findings.

### 4.1. Stress and emotional reactions triggered by simulated nature

Exposure to simulated nature is beneficial for the recovery of perceived stress. According to van den Berg et al. (2003), viewing nature pictures is more likely to reduce perceived stress scores than viewing urban pictures, which may be mediated by a preference for natural environments. An additional study reported that seniors’ perceived stress change scores decreased slightly in nature, despite not reaching conventional statistical significance (Chan et al., 2021b). However, none of the outcome measures characterizing physiological stress supports a consistent difference, which differs from the findings of previous reviews limiting the scope to actual natural environments (Mygind et al., 2019; Yao et al., 2021). Overemphasizing visual exposure and disregarding other pathways might account for it. Numerous studies have shown that physiological recovery cannot be merely attributed to a visual phenomenon (Nukarinen et al., 2022). Plausible pathways linking actual natural environments to physiological states, such as promoting immune function, releasing high concentrations of negative air ions, and effecting microorganisms living on the skin and in the gut (Kuo, 2015), are rarely mentioned in most simulated nature studies.

The current review, in agreement with a previous meta-analysis (McMahan and Estes, 2015), found that the benefits of simulated nature on emotional arousal were observed mainly for positive affect rather than negative affect. Considering the Broaden-and-Build Theory of Positive Emotions may help explain this result. Positive affect is not typically generated in the context of exposure to a life-threatening situation; rather, negative affect induces specific action tendencies in response to survival threats throughout human evolution, such as fight and escape (Fredrickson, 2001). Nature’s characteristics, such as concealment and unobstructed views, help satiate the psychological need to avoid threats, enabling individuals to restore their sense of well-being (Appleton, 1996). The simulation of nature, however, is no more reason to reduce the mobilization of negative affect than a control scene since the simulated processing of nature may cause survival threats to disappear or make it difficult for them to be perceived as threatening. In recorded simulated material, subjects could have difficulty believing that their behavior of seeking out sites with the above characteristics makes sense. Discussions about the level of immersion in a live stream and a recorded video provide some form of support for this conjecture. Presenting the occurring event to the viewer may create a sense of connection to the landscape (Friedman et al., 2008); the latter, on the other hand, makes people stray from it (Kjellgren and Buhrkall, 2010). Computer-generated nature is often portrayed as a “utopia,” selectively presenting the pleasurable and non-aggressive aspects of actual nature. Even if simulated nature does not diminish subject perceptions of threat, its ability to provide psychological security to individuals remains doubtful unless some functional cues in the scene are implied to the subjects.

Although fewer studies were included in the meta-analysis of each relatively specific emotional response, the results still confirm the effectiveness of the simulated nature in improving emotional states. Actual natural environments have been proven to help individuals escape from depression (Roberts et al., 2019; Wang et al., 2019) and anxiety (Zhang et al., 2022). If more evidence demonstrates that these effects are also present in simulated nature, it could serve as a “green narrow-spectrum antibiotic” for treating specific psychological disorders and as a preventive measure for mentally sub-healthy people. The threshold representing optimal efficacy can be determined by conducting dose–response experiments, taking advantage of the unique methodological advantages of simulated natural studies in reducing the uncontrollable factor (Blascovich et al., 2002; Roberts et al., 2019; Browning M. H. E. M. et al., 2020). Researchers should target specific clinical populations according to the specific emotional concepts they would like to explore. In addition, biomedical techniques for measurements of central nervous system activity, such as functional near-infrared spectroscopy (fNIRS) and functional magnetic resonance imaging (fMRI), will further our understanding of the ecological validity of the simulated nature. Some work has found their potential in revealing the neurobiological basis of affective control (Kim et al., 2010; Zhang Z. et al., 2020; Yamashita et al., 2021).

Research conducted online allows researchers to examine the restorative effects of simulated nature on a wider range of groups with diverse social backgrounds. As a result, the current limitation of lack of external validity due to the predominance of students in the subjects will be overcome (Hanel and Vione, 2016; Browning M. H. E. M. et al., 2020; Riches et al., 2021). Conversely, difficulties in supervising the protocol execution and controlling immersion levels may have contributed to the creation of more confounding factors than offline experiments. Further studies are needed to elucidate whether the channel for implementation may modulate emotional and stress responses.

### 4.2. Immersion and restorative effect

The current review suggested that medium immersion resulted in a large effect of positive affect improvement, whereas low and high immersion produced nearly identical small effects. Experiencing high immersion may make people feel uncomfortable, especially if they experience cybersickness (Sharples et al., 2008; Browning M. H. E. M. et al., 2020). Additionally, the proportion of limb movements in the simulation might be related to the severity of cybersickness. According to sensory conflict theory, actual displacement information provided by the vestibular system can be differs from what the visual system perceived (LaViola, 2000). In this way, subjects’ responses to simulated nature are not only a reflection of their subconscious biophilia (Kellert and Wilson, 1993; Wilson, 2021) but also the outcome of a more sophisticated process of judging the rationality of audio-visual information. Depleting directed attention in interference control may somewhat diminish the restorative effects of immersion in simulated nature (Kaplan, 1995). It should be noted that the affect changes caused by cybersickness may be mistakenly attributed to the environment (Van der Spek and Houtkamp, 2008). Future experiments should strive to enhance the real-time performance of motion capture to create a realistic motion parallax effect. Moreover, sufficiently evaluating the single exposure duration in a pilot study is essential. Researchers are encouraged to include cybersickness as a separate item in their eligibility screening and to detail the discomfort during and after the exposure.

The presence a user feels in a simulated environment is a necessary mediator for triggering real emotions in simulated environments (Parsons and Rizzo, 2008; Price et al., 2011). Therefore, close attention should be paid to all factors that can effectively translate increased immersion levels into a stronger sense of presence. Awe induced by the vast natural environment may be an effective way to enhance presence. According to Chirico and colleagues, immersive nature videos were more likely to evoke awe than 2D screen videos because the immersive view enhances the sense of physical space and vastness (Chirico et al., 2017a,b; Chirico and Gaggioli, 2019). Immersive scenarios can realistically represent the physical spatial features needed to arouse emotion, implying that the technical limitations preventing people from resonating with the emotions conveyed by nature scenes are being broken down.

Besides, efforts also should be made to consider how to select, establish and maintain immersion. Motion capture patterns are sometimes naturally implied by some scene content or camera movement trajectory. For example, simulated natural scenes with a definite sense of dynamism and axiality are associated with riding (Duncan et al., 2014; Wooller et al., 2018) or driving behaviors (Cackowski and Nasar, 2016; Jiang et al., 2020), but in pavilions, simply sitting and resting is relaxing enough (Luo et al., 2022). A highly immersive VR experience may not be desirable for people with low technology acceptability (Miller et al., 2014; Liu et al., 2020). Furthermore, the experimental design should minimize disruption of the immersion continuum. Individuals’ attention will be diverted from the content if they switch between scenes with different levels of immersion. Ideally, the various experimental sessions should be incorporated into a simulation procedure distributed on the timeline. Simulations of several sessions have been attempted in several studies (Annerstedt et al., 2013; Schebella et al., 2019). This approach also offers the benefit of subject learning and adapting to VR, minimizing the interference of novelty effects (Merchant et al., 2014; Hopp and Gangadharbatla, 2016), aka novelty bias (Riches et al., 2021). People’s curiosity about VR technology was considered to possibly offset any potential relaxing effects (Reece et al., 2022).

Another concern emerging from the review is the limitation of biomedical measurement techniques to limb movement in a highly immersive environment. Some studies state that the reason for not allowing subjects to perform activities is the inability to avoid motion artifacts from physiological recordings during this process (van den Berg et al., 2015; Zhang Z. et al., 2020; Kimura et al., 2021; Filo and Janousek, 2022). Combining emotion recognition technologies in artificial intelligence (e.g., micro-expression recognition) with projection devices may make a difference and promises to minimize the presence of wearable devices in an immersive experience.

A considerable body of research has shown that simulating nature relieves stress and arouses emotions instantly. However, the only study in the review to include repeated exposure yielded an intriguing outcome. It noted that viewing nature videos for short periods each day led to a perceived relaxation in people placed on prolonged lockdown to control COVID-19, but the immediate benefits did not last a week (Zabini et al., 2020). Nature’s effect on health and cognitive improvement may rely on repeated exposure to specific environments or locations (Browning and Alvarez, 2020). However, it remains unclear whether repeated exposure to simulated nature may have positive outcomes in the long run. Changes in mental health status that take a long time to reflect on will likely be overlooked if only data collected shortly after exposure is considered. Including follow-up results may lead to various revisions of the current meta-analysis results. Subjects may feel a deeper and longer-lasting sense of well-being in “post-cognitive” affective responses (Ulrich, 1983) but are also at risk of a negative evaluation due to the after-effects of cybersickness (Van der Spek and Houtkamp, 2008; Somrak et al., 2019). Cybersickness does not always occur immediately after exposure but may last from a few hours to several days, depending on the individual (LaViola, 2000). In addition, because of the lack of convincing evidence to support the persistence of novelty effects, the attractiveness of the high immersion setting itself does not fit as a part of the restorative effect triggered by simulated nature. Collectively, there is a need to repeat exposure experiments with a longer period and follow-ups under high immersion conditions.

### 4.3. Strength and limitation

Combining the bibliographic records of six databases, the fully transparent and reproducible systematic review and meta-analysis included more than 20 outcome measures. Thus, it provides a comprehensive and realistic synthesis of evidence for the stress and emotional reactions triggered by simulated nature. A key strength is that we observed the effects of immersion levels on positive and negative affect through subgroup analyses, which will serve as a reference for future research when presenting simulated nature experiences.

Some limitations exist in this review. The gray literature was excluded, which may result in missing unpublished data. Methodological heterogeneity among the studies may make it difficult to interpret the findings of this review due to numerous confounding factors. Thus, strict inclusion and exclusion criteria were developed to improve the comparability of the included literature. Despite the subgroup analysis is pre-specified rather than *post hoc*, it is observational in nature (Deeks et al., 2019). Further empirical studies will enable a more accurate picture of how the immersion level of simulated environments works.

Another limitation of this review is that more technical details related to immersion, such as screen size (de Kort et al., 2006), perceived distance (Souza et al., 2015), field-of-view (Schoor et al., 2010), have not been discussed. The lack of technical features in articles restricts a deeper analysis of immersion levels. A detailed description of the equipment parameters and site layout will serve to reproduce and compare the restorative effects of simulated nature.

Furthermore, we could not exclude any studies based on low quality, as all included studies had a moderate to high ROB. Failure to standardize the implementation or reporting of methods and results may hamper the extrapolation and interpretation of our findings.

### 4.4. Future studies

The role of nature simulation in assisting individuals to improve their psychological well-being must be considered more specifically and cautiously. A discussion of high immersion’s facilitative or inhibitive restorative effects may be appropriate only with a clear definition of the study purpose, the subjects, and the simulation method. Policymakers should be vigilant about the intent to generalize the health benefits of nature based solely on results under specific conditions (Bowler et al., 2010). A more constructive perspective is that actual nature and simulated nature hold promise as complementary solutions to address human mental health issues jointly. Several recent simulated natural short-term exposure experiments conducted for frontline healthcare workers have expressed researchers’ concerns about the specific conditions applicable to prescriptions for simulated nature (Putrino et al., 2020; Pallavicini et al., 2021; Beverly et al., 2022). Future public health policy will profit from efforts to adequately integrate the mental health benefits of actual and simulated nature.

## 5. Conclusion

This systematic review and meta-analysis demonstrate that exposure to simulated nature contributes to stress recovery and emotional arousal, and the immersion level may moderate the restorative effect. Simply applying the mindset of actual nature studies may lead us to overlook critical antecedent issues related to the technical characteristics of simulated nature. This will plague subsequent empirical studies and will not be supportive in developing a theoretical framework more applicable to explain the mental health benefits of simulated nature. We call for more attention to the technical features of simulated nature, including immersion, and their mechanisms of action to elucidate the association between simulated nature and psychological well-being.

## Data availability statement

The original contributions presented in the study are included in the article/Supplementary material, further inquiries can be directed to the corresponding author.

## Author contributions

HL: conceptualization, methodology, investigation, formal analysis, writing – original draft, and writing – review and editing. YX: methodology, investigation, formal analysis, and writing – review and editing. BZ: formal analysis and writing – review and editing. WW: writing – review and editing. All authors contributed to the article and approved the submitted version.

## Conflict of interest

The authors declare that the research was conducted in the absence of any commercial or financial relationships that could be construed as a potential conflict of interest.

## Publisher’s note

All claims expressed in this article are solely those of the authors and do not necessarily represent those of their affiliated organizations, or those of the publisher, the editors and the reviewers. Any product that may be evaluated in this article, or claim that may be made by its manufacturer, is not guaranteed or endorsed by the publisher.

## Supplementary material

The Supplementary material for this article can be found online at: https://www.frontiersin.org/articles/10.3389/fpsyg.2022.1058177/full#supplementary-material

SUPPLEMENTARY FIGURE S1 Cochrane risk of Bias 2.0 table.

SUPPLEMENTARY FIGURE S2 Subgroup analysis of the effects of simulated nature exposure on negative affect.

SUPPLEMENTARY FIGURE S3 Subgroup analysis of the effects of simulated nature exposure on tension.

SUPPLEMENTARY FIGURE S4 Subgroup analysis of the effects of simulated nature exposure on vigor.

SUPPLEMENTARY FIGURE S5 Subgroup analysis of the effects of simulated nature exposure on calmness.

SUPPLEMENTARY FIGURE S6 Funnel plot of positive affect.

SUPPLEMENTARY FIGURE S7 Funnel plot of negative affect.
