# Supplementary figures and images for: Effects of immersion in a simulated natural environment on stress reduction and emotional arousal: A systematic review and meta-analysis
Source: Front Psychol. 2023 Jan 9;13:1058177. doi: 10.3389/fpsyg.2022.1058177 (PMC9869155; doi:10.3389/fpsyg.2022.1058177)

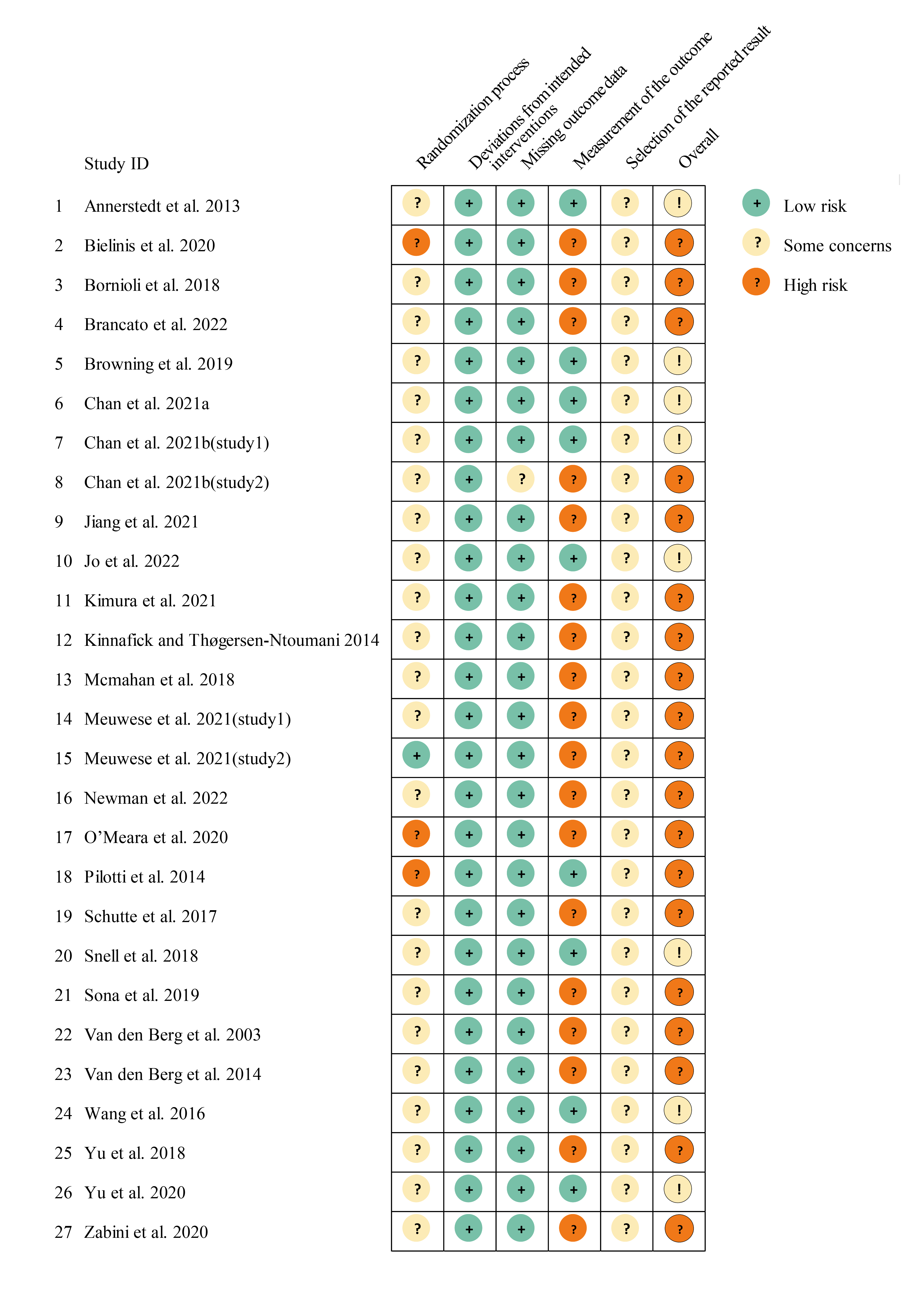

Supplement: SUPPLEMENTARY FIGURE S1 — Cochrane risk of Bias 2.0 table. [file Image_1.TIF]

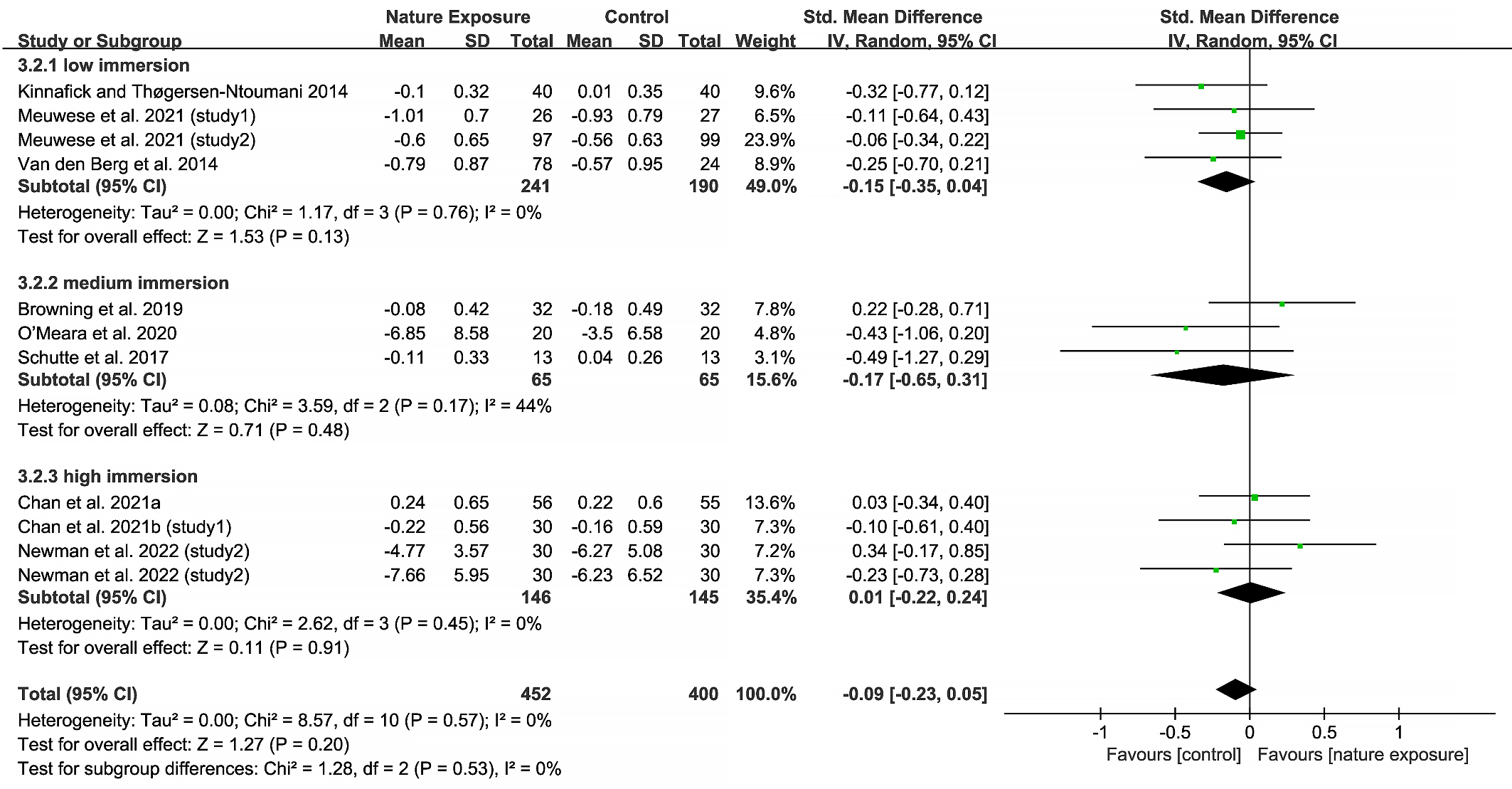

Supplement: SUPPLEMENTARY FIGURE S2 — Subgroup analysis of the effects of simulated nature exposure on negative affect. [file Image_2.TIF]

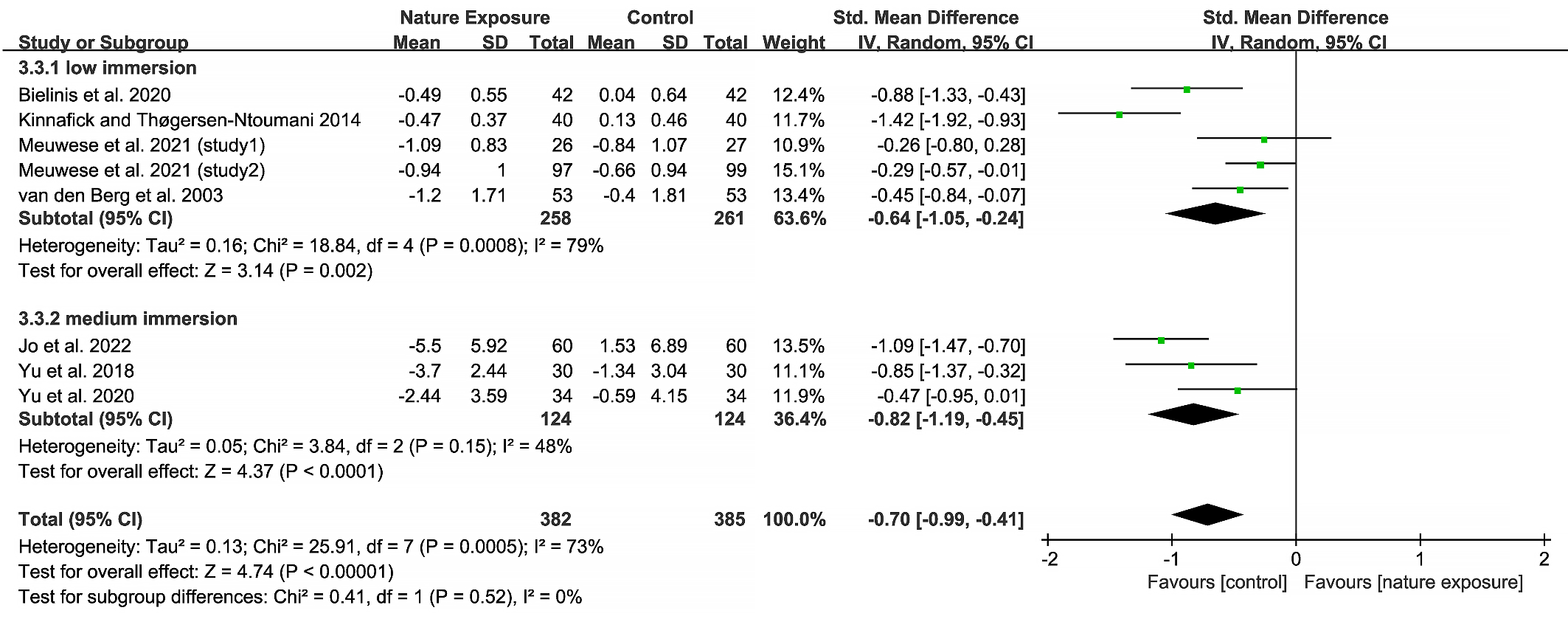

Supplement: SUPPLEMENTARY FIGURE S3 — Subgroup analysis of the effects of simulated nature exposure on tension. [file Image_3.TIF]

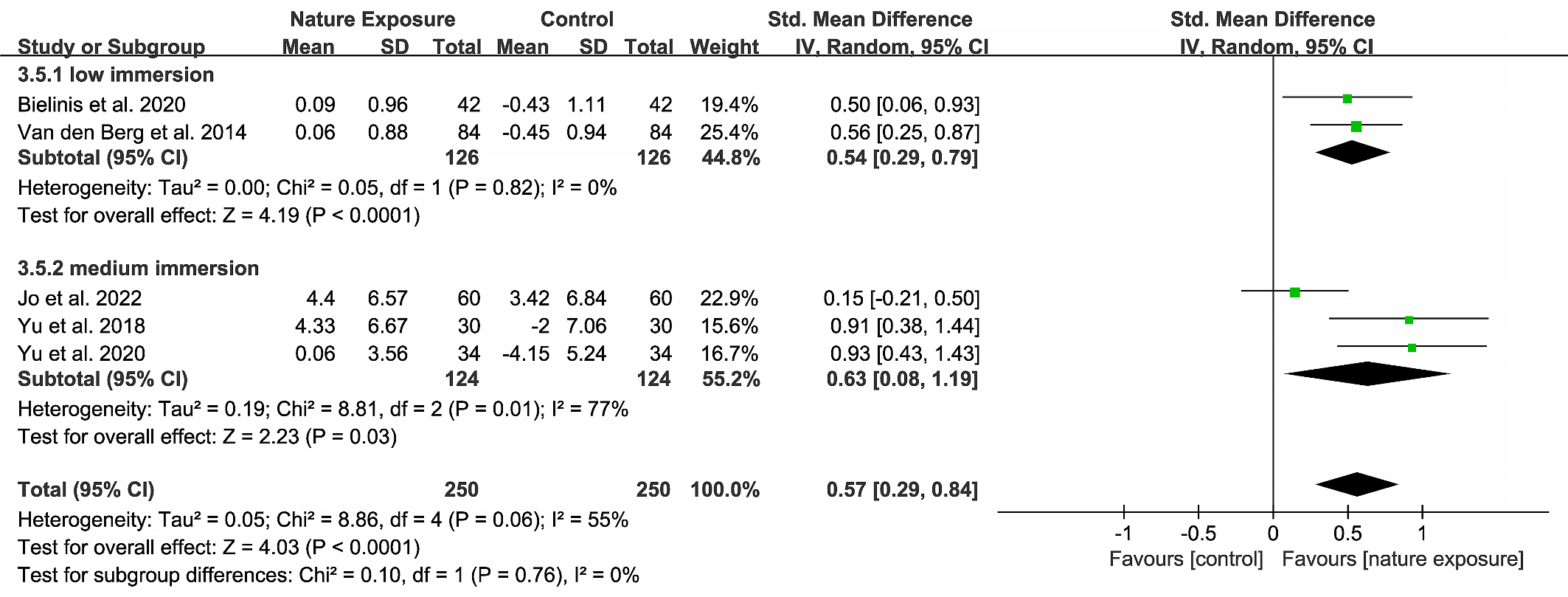

Supplement: SUPPLEMENTARY FIGURE S4 — Subgroup analysis of the effects of simulated nature exposure on vigor. [file Image_4.TIF]

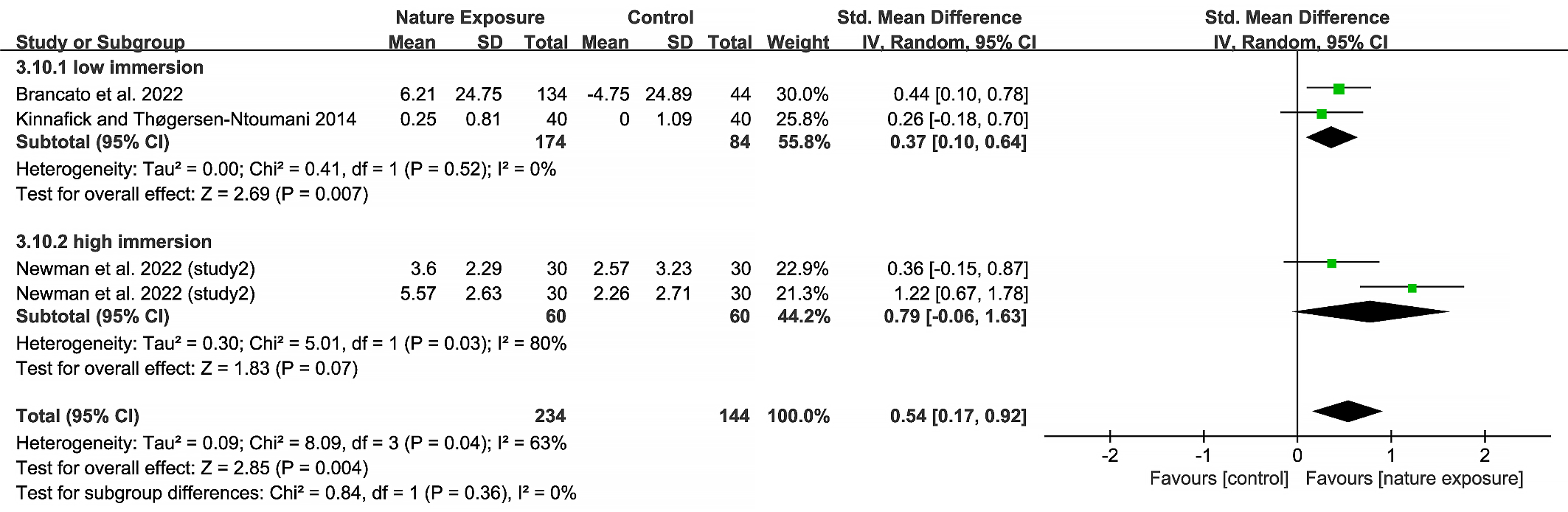

Supplement: SUPPLEMENTARY FIGURE S5 — Subgroup analysis of the effects of simulated nature exposure on calmness. [file Image_5.TIF]

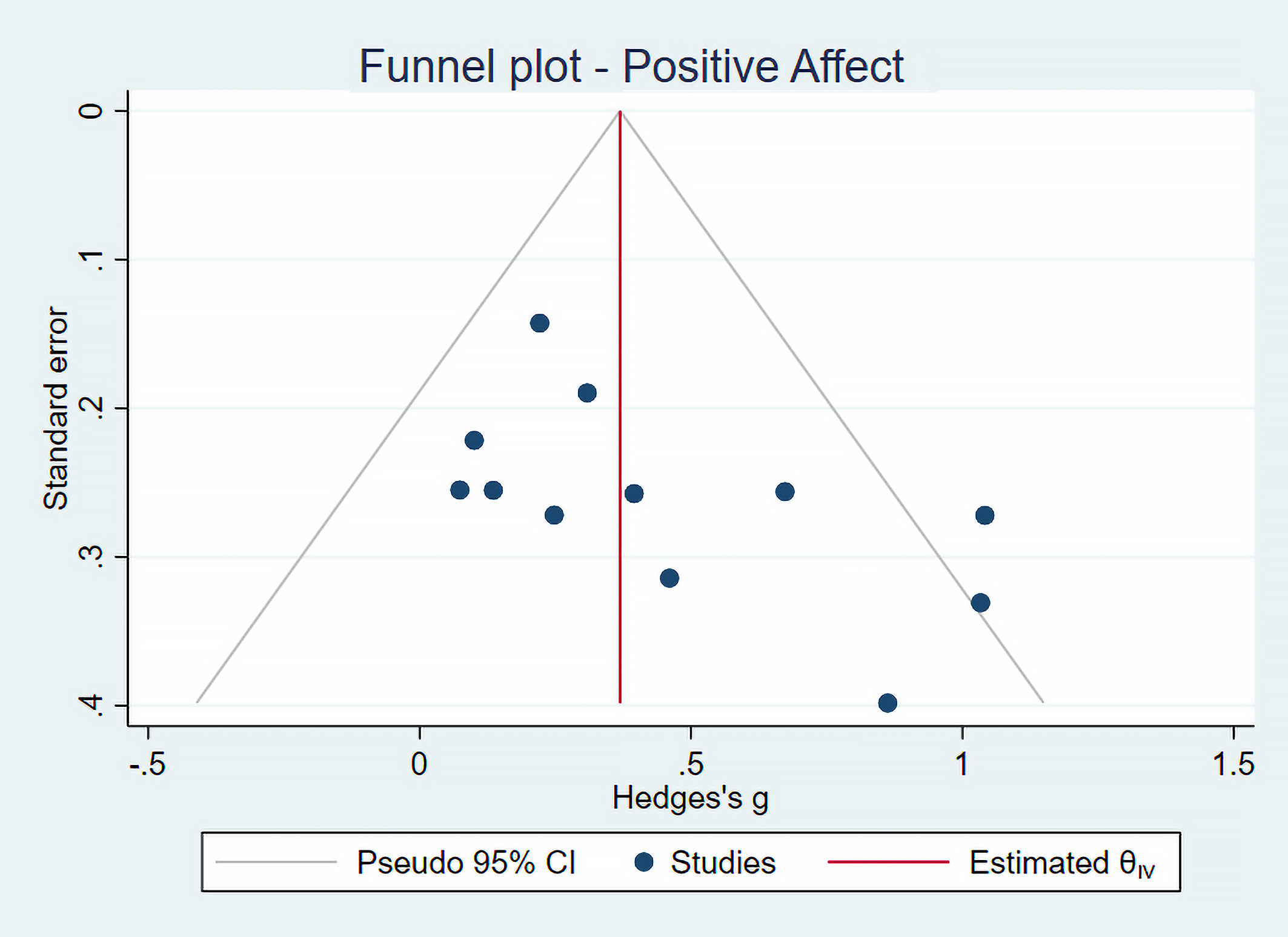

Supplement: SUPPLEMENTARY FIGURE S6 — Funnel plot of positive affect. [file Image_6.TIF]

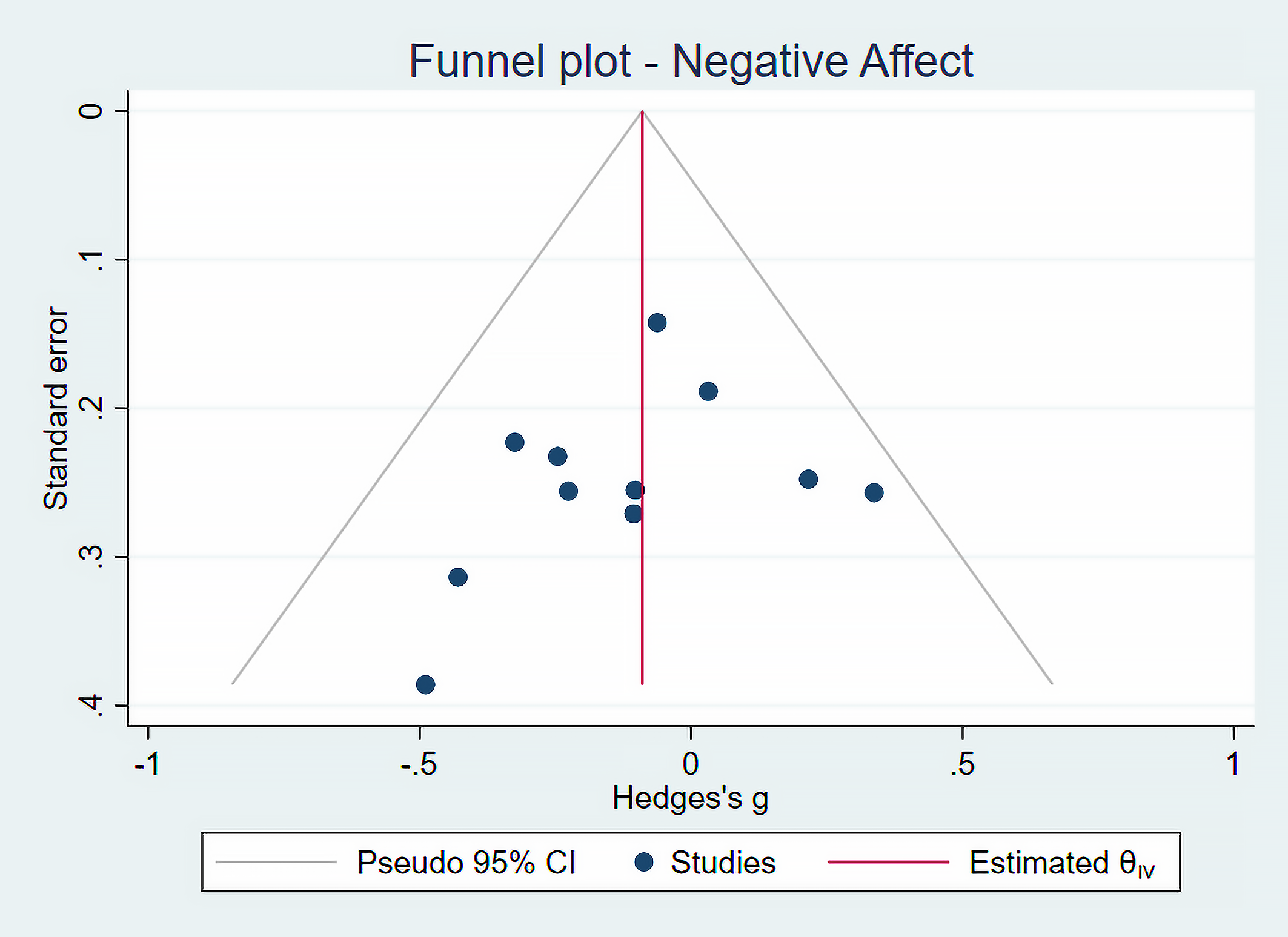

Supplement: SUPPLEMENTARY FIGURE S7 — Funnel plot of negative affect. [file Image_7.TIF]
