# Supplementary material for: Effects of immersion in a simulated natural environment on stress reduction and emotional arousal: A systematic review and meta-analysis
Source: Front Psychol. 2023 Jan 9;13:1058177. doi: 10.3389/fpsyg.2022.1058177 (PMC9869155; doi:10.3389/fpsyg.2022.1058177)
Supplement: Supplementary file 8 [file Table_1.DOCX]

Supplementary Material

**Table of contents**

[Table S1. PRISMA 2020 checklist 3](#_Toc119375847)

[Table S2. Search strategies for electronic databases 6](#_Toc119375848)

[Table S3. Summary results for all included studies 9](#_Toc119375849)

[Table S4. ROBINS-I assessments 16](#_Toc119375850)

[Table S5. Interpretation of the main outcomes 16](#_Toc119375851)

[Table S6. Sensitivity analysis of the effects on positive affect 17](#_Toc119375852)

[Table S7. Sensitivity analysis of the effects on negative affect 17](#_Toc119375853)

[Table S8. Sensitivity analysis of the effects on perceived stress 17](#_Toc119375854)

[Table S9. Sensitivity analysis of the effects on total mood disturbance (TMD) 17](#_Toc119375855)

[Table S10. Sensitivity analysis of the effects on tension 18](#_Toc119375856)

[Table S11. Sensitivity analysis of the effects on fatigue 18](#_Toc119375857)

[Table S12. Sensitivity analysis of the effects on vigor 18](#_Toc119375858)

[Table S13. Sensitivity analysis of the effects on anxiety 18](#_Toc119375859)

[Table S14. Sensitivity analysis of the effects on depression 19](#_Toc119375860)

[Table S15. Sensitivity analysis of the effects on confusion 19](#_Toc119375861)

[Table S16. Sensitivity analysis of the effects on anger 19](#_Toc119375862)

[Table S17. Sensitivity analysis of the effects on happiness 19](#_Toc119375863)

[Table S18. Sensitivity analysis of the effects on calmness 19](#_Toc119375864)

[Table S19. Sensitivity analysis of the effects on arousal 19](#_Toc119375865)

[Table S20. Sensitivity analysis of the effects on valence 20](#_Toc119375866)

[Table S21. Sensitivity analysis of the effects on systolic blood pressure (SBP) 20](#_Toc119375867)

[Table S22. Sensitivity analysis of the effects on diastolic blood pressure (DBP) 20](#_Toc119375868)

[Table S23. Sensitivity analysis of the effects on heart rate (HR) 20](#_Toc119375869)

[Table S24. Sensitivity analysis of the effects on low frequency power (LF power) 20](#_Toc119375870)

[Table S25. Sensitivity analysis of the effects on high frequency power (HF power) 20](#_Toc119375871)

[Table S26. Sensitivity analysis of the effects on low frequency/high frequency (LF/HF) 21](#_Toc119375872)

[Table S27. Sensitivity analysis of the effects on root mean square on successive differences (RMSSD) 21](#_Toc119375873)

[Table S28. Summary of Findings table 22](#_Toc119375874)

[Reference 24](#_Toc119375875)

# Table S1. PRISMA 2020 checklist

| **Section and Topic** | **Item #** | **Checklist item** | **Location where item is reported** |
| --- | --- | --- | --- |
| **TITLE** | | |  |
| Title | 1 | Identify the report as a systematic review. | 1 |
| **ABSTRACT** | | |  |
| Abstract | 2 | See the PRISMA 2020 for Abstracts checklist. | 1 |
| **INTRODUCTION** | | |  |
| Rationale | 3 | Describe the rationale for the review in the context of existing knowledge. | 2 |
| Objectives | 4 | Provide an explicit statement of the objective(s) or question(s) the review addresses. | 2 – 3 |
| **METHODS** | | |  |
| Eligibility criteria | 5 | Specify the inclusion and exclusion criteria for the review and how studies were grouped for the syntheses. | 3 |
| Information sources | 6 | Specify all databases, registers, websites, organisations, reference lists and other sources searched or consulted to identify studies. Specify the date when each source was last searched or consulted. | 3 |
| Search strategy | 7 | Present the full search strategies for all databases, registers and websites, including any filters and limits used. | 3 |
| Selection process | 8 | Specify the methods used to decide whether a study met the inclusion criteria of the review, including how many reviewers screened each record and each report retrieved, whether they worked independently, and if applicable, details of automation tools used in the process. | 3 |
| Data collection process | 9 | Specify the methods used to collect data from reports, including how many reviewers collected data from each report, whether they worked independently, any processes for obtaining or confirming data from study investigators, and if applicable, details of automation tools used in the process. | 3 – 4 |
| Data items | 10a | List and define all outcomes for which data were sought. Specify whether all results that were compatible with each outcome domain in each study were sought (e.g. for all measures, time points, analyses), and if not, the methods used to decide which results to collect. | 4 – 5 |
|  | 10b | List and define all other variables for which data were sought (e.g. participant and intervention characteristics, funding sources). Describe any assumptions made about any missing or unclear information. | 5 |
| Study risk of bias assessment | 11 | Specify the methods used to assess risk of bias in the included studies, including details of the tool(s) used, how many reviewers assessed each study and whether they worked independently, and if applicable, details of automation tools used in the process. | 4 |
| Effect measures | 12 | Specify for each outcome the effect measure(s) (e.g. risk ratio, mean difference) used in the synthesis or presentation of results. | 4 – 5 |
| Synthesis methods | 13a | Describe the processes used to decide which studies were eligible for each synthesis (e.g. tabulating the study intervention characteristics and comparing against the planned groups for each synthesis (item #5)). | 5 |
|  | 13b | Describe any methods required to prepare the data for presentation or synthesis, such as handling of missing summary statistics, or data conversions. | 5 |
|  | 13c | Describe any methods used to tabulate or visually display results of individual studies and syntheses. | 5 |
|  | 13d | Describe any methods used to synthesize results and provide a rationale for the choice(s). If meta-analysis was performed, describe the model(s), method(s) to identify the presence and extent of statistical heterogeneity, and software package(s) used. | 5 |
|  | 13e | Describe any methods used to explore possible causes of heterogeneity among study results (e.g. subgroup analysis, meta-regression). | 5 |
|  | 13f | Describe any sensitivity analyses conducted to assess robustness of the synthesized results. | 5 |
| Reporting bias assessment | 14 | Describe any methods used to assess risk of bias due to missing results in a synthesis (arising from reporting biases). | 4 |
| Certainty assessment | 15 | Describe any methods used to assess certainty (or confidence) in the body of evidence for an outcome. | 4 |
| **RESULTS** | | |  |
| Study selection | 16a | Describe the results of the search and selection process, from the number of records identified in the search to the number of studies included in the review, ideally using a flow diagram. | 5 |
|  | 16b | Cite studies that might appear to meet the inclusion criteria, but which were excluded, and explain why they were excluded. | 5 |
| Study characteristics | 17 | Cite each included study and present its characteristics. | 5 |
| Risk of bias in studies | 18 | Present assessments of risk of bias for each included study. | 7 |
| Results of individual studies | 19 | For all outcomes, present, for each study: (a) summary statistics for each group (where appropriate) and (b) an effect estimate and its precision (e.g. confidence/credible interval), ideally using structured tables or plots. | 9 |
| Results of syntheses | 20a | For each synthesis, briefly summarise the characteristics and risk of bias among contributing studies. | 10 |
|  | 20b | Present results of all statistical syntheses conducted. If meta-analysis was done, present for each the summary estimate and its precision (e.g. confidence/credible interval) and measures of statistical heterogeneity. If comparing groups, describe the direction of the effect. | 5 – 9 |
|  | 20c | Present results of all investigations of possible causes of heterogeneity among study results. | 8 |
|  | 20d | Present results of all sensitivity analyses conducted to assess the robustness of the synthesized results. | 8 |
| Reporting biases | 21 | Present assessments of risk of bias due to missing results (arising from reporting biases) for each synthesis assessed. | 9 – 10 |
| Certainty of evidence | 22 | Present assessments of certainty (or confidence) in the body of evidence for each outcome assessed. | 10 |
| **DISCUSSION** | | |  |
| Discussion | 23a | Provide a general interpretation of the results in the context of other evidence. | 10 – 13 |
|  | 23b | Discuss any limitations of the evidence included in the review. | 13 |
|  | 23c | Discuss any limitations of the review processes used. | 13 |
|  | 23d | Discuss implications of the results for practice, policy, and future research. | 13 |
| **OTHER INFORMATION** | | |  |
| Registration and protocol | 24a | Provide registration information for the review, including register name and registration number, or state that the review was not registered. | 3 |
|  | 24b | Indicate where the review protocol can be accessed, or state that a protocol was not prepared. | 3 |
|  | 24c | Describe and explain any amendments to information provided at registration or in the protocol. | 3 |
| Support | 25 | Describe sources of financial or non-financial support for the review, and the role of the funders or sponsors in the review. | 14 |
| Competing interests | 26 | Declare any competing interests of review authors. | 14 |
| Availability of data, code and other materials | 27 | Report which of the following are publicly available and where they can be found: template data collection forms; data extracted from included studies; data used for all analyses; analytic code; any other materials used in the review. | 13 – 14 |

*From:*  Page MJ, McKenzie JE, Bossuyt PM, Boutron I, Hoffmann TC, Mulrow CD, et al. The PRISMA 2020 statement: an updated guideline for reporting systematic reviews. BMJ 2021;372:n71. doi: 10.1136/bmj.n71

For more information, visit: <http://www.prisma-statement.org/>

# Table S2. Search strategies for electronic databases

| **Database** | **Search strategy** |
| --- | --- |
| Web of Science | #1 TS= (“natural environment*” OR “natural area*” OR “natural space*” OR “natural scene*” OR “natural setting*” OR “natural view*” OR “outdoor*” OR “open space*” OR “greenness” OR “greenery” OR “green space*” OR “greenspace” OR “green area*” OR “greening” OR " green environment*" OR “greenway” OR “green belt” OR “green exercise” OR “wilderness” OR “wild land” OR “wild area*” OR “wild space” OR “forest” OR “woodland” OR “natural landscape” OR “park” OR “garden” OR “vegetation” OR “recreation resource” OR “restorative environment*” OR “healing environment*” )  #2 TS= (“virtual real*” OR “virtual-real*” OR “VR” OR “virtual environment*” OR “simulated environment*” OR “virtual natur*” OR “simulated natur*”OR “simulation*” OR “immersi*” OR “photograph*” OR “picture*” OR “video*” OR “film*” OR “display*” OR “media” OR “HMD” OR “visual*” OR “panoramic*”)  #3 TS= (“mental health” OR “emotion*” OR “affective state” OR “affective response” OR “psychologic*” OR “psychophysiologic*”OR “health outcome*” OR “mood” OR “stress” OR “wellbeing” OR “well-being” OR “anxiet*” OR “restoration” OR “restorative*” OR “recovery effect*” OR “relax*”OR “environmental stressor*”)  #4 TS= (“randomized controlled trial” OR “randomized” OR “RCT” OR “control group” OR “non-randomized” OR “crossover” OR “cross-over” OR “intervention” OR “exposure” OR “compare” OR “comparison” OR “pre-post”)  #5 TS= (“participant” OR “student” OR “subject” OR “volunteer” OR “people” OR “respondent” OR “adult” OR “teenager” OR “older” OR “m?n” OR “wom?n” OR “human”)  #6 (#1 AND #2 AND #3 AND #4 AND #5) AND (LA==("ENGLISH") |
| Scopus | ( TITLE-ABS-KEY ( "natural environment*" OR "natural area*" OR "natural space*" OR "natural scene*" OR "natural setting*" OR "natural view*" OR "outdoor*" OR "open space*" OR "greenness" OR "greenery" OR "green space*" OR "greenspace" OR "green area*" OR "greening" OR " green environment*" OR "greenway" OR "green belt" OR "green exercise" OR "wilderness" OR "wild land" OR "wild area*" OR "wild space" OR "forest" OR "woodland" OR "natural landscape" OR "park" OR "garden" OR "vegetation" OR "recreation resource" OR "restorative environment*" OR "healing environment*" ) ) AND ( TITLE-ABS-KEY ( "virtual real*" OR "virtual-real*" OR "VR" OR "virtual environment*" OR "simulated environment*" OR "virtual natur*" OR "simulated natur*" OR "simulation*" OR "immersi*" OR "photograph*" OR "picture*" OR "video*" OR "film*" OR "media" OR "desktop*" OR "HMD" OR "visual*" OR "panoramic*" ) ) AND ( TITLE-ABS-KEY ( "mental health" OR "emotion*" OR "affective state" OR "affective response" OR "psychologic*" OR "psychophysiologic*" OR "health outcome*" OR "mood" OR "stress" OR "wellbeing" OR "well-being" OR "anxiet*" OR "restoration" OR "restorative*" OR "recovery effect*" OR "relax*" OR "environmental stressor*" ) ) AND ( TITLE-ABS-KEY ( "randomized controlled trial" OR "randomized" OR "RCT" OR "control group" OR "non-randomized" OR "crossover" OR "cross-over" OR "intervention" OR "exposure" OR "compare" OR "comparison" OR "pre-post" ) ) AND ( TITLE-ABS-KEY ( "participant" OR "student" OR "subject" OR "volunteer" OR "people" OR "respondent" OR "adult" OR "teenager" OR "older" OR "m?n" OR "wom?n" OR "human" ) ) AND ( LIMIT-TO ( LANGUAGE , "English" ) ) |
| PsycINFO | S1 SU “natural environment*” OR “natural area*” OR “natural space*” OR “natural scene*” OR “natural setting*” OR “natural view*” OR “outdoor*” OR “open space*” OR “greenness” OR “greenery” OR “green space*” OR “greenspace” OR “green area*” OR “greening” OR " green environment*" OR “greenway” OR “green belt” OR “green exercise” OR “wilderness” OR “wild land” OR “wild area*” OR “wild space” OR “forest” OR “woodland” OR “natural landscape” OR “park” OR “garden” OR “vegetation” OR “recreation resource” OR “restorative environment*” OR “healing environment*”  S2 SU “virtual real*” OR “virtual-real*” OR “VR” OR “virtual environment*” OR “simulated environment*” OR “virtual natur*” OR “simulated natur*”OR “simulation*” OR “immersi*” OR “photograph*” OR “picture*” OR “video*” OR “film*” OR “display*” OR “media” OR “HMD” OR “visual*” OR “panoramic*”  S3 SU “mental health” OR “emotion*” OR “affective state” OR “affective response” OR “psychologic*” OR “psychophysiologic*”OR “health outcome*” OR “mood” OR “stress” OR “wellbeing” OR “well-being” OR “anxiet*” OR “restoration” OR “restorative*” OR “recovery effect*” OR “relax*”OR “environmental stressor*”  S4 SU “randomized controlled trial” OR “randomized” OR “RCT” OR “control group” OR “non-randomized” OR “crossover” OR “cross-over” OR “intervention” OR “exposure” OR “compare” OR “comparison” OR “pre-post”  S5 AB “participant” OR “student” OR “subject” OR “volunteer” OR “people” OR “respondent” OR “adult” OR “teenager” OR “older” OR “m?n” OR “wom?n” OR “human”  S6 S1 AND S2 AND S3 AND S4 AND S5 |
| PubMed | #1 “natural environment*” OR “natural area*” OR “natural space*” OR “natural scene*” OR “natural setting*” OR “natural view*” OR “outdoor*” OR “open space*” OR “greenness” OR “greenery” OR “green space*” OR “greenspace” OR “green area*” OR “greening” OR " green environment*" OR “greenway” OR “green belt” OR “green exercise” OR “wilderness” OR “wild land” OR “wild area*” OR “wild space” OR “forest” OR “woodland” OR “natural landscape” OR “park” OR “garden” OR “vegetation” OR “recreation resource” OR “restorative environment*” OR “healing environment*”  #2 “virtual real*” OR “virtual-real*” OR “VR” OR “virtual environment*” OR “simulated environment*” OR “virtual natur*” OR “simulated natur*”OR “simulation*” OR “immersi*” OR “photograph*” OR “picture*” OR “video*” OR “film*” OR “display*” OR “media” OR “HMD” OR “visual*” OR “panoramic*”  #3 Stress, Physiological [MeSH Major Topic]  #4 “Abiotic Stress Reaction” OR “Response, Abiotic Stress” OR “Reactions, Abiotic Stress” OR “Abiotic Stress Reactions” OR “Reaction, Abiotic Stress” OR “Abiotic Stress Responses” OR “Abiotic Stress Response” OR “Physiological Stress Response” OR “Stress Reaction, Physiological” OR “Physiological Stress Reaction” OR “Stress Response, Physiological” OR “Physiological Stress Responses” OR “Physiological Stress Reactivity” OR “Physiological Stress Reactions” OR “Stress, Biotic” OR “Biotic Stresses” OR “Biotic Stress OR “Physiological Stresses” OR “Physiological Stress OR “Stress, Metabolic” OR “Metabolic Stress” OR “Metabolic Stresses” OR “Stress, Biological” OR “Biological Stress” OR “Biological Stresses” OR “Abiotic Stresses” OR “Stress, Abiotic” OR “Abiotic Stress” OR “Metabolic Stress Response” OR “Metabolic Stress Responses” OR “Stress Response, Metabolic” OR “Response, Metabolic Stress”  #5 “Stress, Psychological” [MeSH Major Topic]  #6 “Stress, Life” OR “Life Stresses” OR “Psychological Stresses” OR “Stresses, Life” OR “Stress, Psychologic” OR “Life Stress” OR “Stresses, Psychological” OR “Psychologic Stress” OR “Psychological Stress”  #7 #1 AND #2 AND (#3 OR #4 OR #5 OR #6) Filters: English, Humans |
| Cochrane library | #1 (“natural environment*” OR “natural area*” OR “natural space*” OR “natural scene*” OR “natural setting*” OR “natural view*” OR “outdoor*” OR “open space*” OR “greenness” OR “greenery” OR “green space*” OR “greenspace” OR “green area*” OR “greening” OR " green environment*" OR “greenway” OR “green belt” OR “green exercise” OR “wilderness” OR “wild land” OR “wild area*” OR “wild space” OR “forest” OR “woodland” OR “natural landscape” OR “park” OR “garden” OR “vegetation” OR “recreation resource” OR “restorative environment*” OR “healing environment*”):ti,ab,kw  #2 (“virtual real*” OR “simulat*” OR “immersi*” OR “photograph*” OR “picture*” OR “video*” OR “film*” OR “image*” OR “display*” OR “media” OR “desktop*” OR “cave*” OR “HMD” OR “visual*” OR “panoramic*”):ti,ab,kw (Word variations have been searched)  #3 MeSH descriptor: [Stress, Physiological] explode all trees  #4 (“Abiotic Stress Reaction” OR “Response, Abiotic Stress” OR “Reactions, Abiotic Stress” OR “Abiotic Stress Reactions” OR “Reaction, Abiotic Stress” OR “Abiotic Stress Responses” OR “Abiotic Stress Response” OR “Physiological Stress Response” OR “Stress Reaction, Physiological” OR “Physiological Stress Reaction” OR “Stress Response, Physiological” OR “Physiological Stress Responses” OR “Physiological Stress Reactivity” OR “Physiological Stress Reactions” OR “Stress, Biotic” OR “Biotic Stresses” OR “Biotic Stress OR “Physiological Stresses” OR “Physiological Stress OR “Stress, Metabolic” OR “Metabolic Stress” OR “Metabolic Stresses” OR “Stress, Biological” OR “Biological Stress” OR “Biological Stresses” OR “Abiotic Stresses” OR “Stress, Abiotic” OR “Abiotic Stress” OR “Metabolic Stress Response” OR “Metabolic Stress Responses” OR “Stress Response, Metabolic” OR “Response, Metabolic Stress”):ti,ab,kw  #5 MeSH descriptor: [Stress, Psychological] explode all trees  #6 (“Stress, Life” OR “Life Stresses” OR “Psychological Stresses” OR “Stresses, Life” OR “Stress, Psychologic” OR “Life Stress” OR “Stresses, Psychological” OR “Psychologic Stress” OR “Psychological Stress”):ti,ab,kw  #7 (#1 AND #2 AND (#3 OR #4 OR #5 OR #6)) NOT "animal" in Trials |
| Embase | #1 ‘natural environment*’ OR ‘natural area*’ OR ‘natural space*’ OR ‘natural scene*’ OR ‘natural setting*’ OR ‘natural view*’ OR ‘outdoor*’ OR ‘open space*’ OR ‘greenness’ OR ‘greenery’ OR ‘green space*’ OR ‘greenspace’ OR ‘green area*’ OR ‘greening’ OR ‘green environment*’ OR ‘greenway*’ OR ‘green belt’ OR ‘green exercise’ OR ‘wilderness’ OR ‘wild land’ OR ‘wild area*’ OR ‘wild space’ OR ‘forest’ OR ‘woodland’ OR ‘natural landscape’ OR ‘park’ OR ‘garden’ OR ‘vegetation’ OR ‘recreation resource’ OR ‘restorative environment*’ OR ‘healing environment*’  #2 ‘virtual real*’ OR ‘virtual-real*’ OR ‘VR’ OR ‘virtual environment*’ OR ‘simulated environment*’ OR ‘virtual natur*’ OR ‘simulated natur*’ OR ‘simulation*’ OR ‘immersi*’ OR ‘photograph*’ OR ‘picture*’ OR ‘video*’ OR ‘film*’ OR ‘display*’ OR ‘media’ OR ‘HMD’ OR ‘visual*’ OR ‘panoramic’  #3 'physiological stress'/exp  #4 ‘alarm reaction’ OR ‘biologic stress’ OR ‘biological stress’ OR ‘organismal stress’ OR ‘physiologic stress’ OR ‘physiological stresses’ OR ‘stress’ OR ‘stress capacity’ OR ‘stress reaction’ OR ‘stress resistance’ OR ‘stress response’ OR ‘stress situation’ OR ‘stress tolerance’ OR ‘stress, physiological’  #5 ‘mental stress’/exp  #6 ‘mental stresses’ OR ‘mental tension’ OR ‘nervous stress’ OR ‘psychic stress’ OR ‘psychic tension’ OR ‘psycho-social stress’ OR ‘psycho-social stresses’ OR ‘psychologic stress’ OR ‘psychological stress’ OR ‘psychosocial stress’ OR ‘psychosocial stresses’ OR ‘stress’ OR ‘mental’ OR ‘stress, psychologic’ OR ‘stress, psychological’ OR ‘tension’ OR ‘mental’ OR ‘tension, psychic’  #7 ‘randomized controlled trial’/exp OR ‘randomized controlled trial’ OR ‘randomized’ OR ‘RCT’ OR 'controlled study'/exp OR ‘control group’ OR ‘non-randomized’ OR ‘crossover’ OR ‘cross-over’ OR ‘intervention’ OR ‘exposure’ OR ‘comparison’ OR ‘compare’ OR ‘pre-post’  #8 #1 AND #2 AND (#3 OR #4 OR #5 OR #6) AND #7 AND [article]/lim AND [humans]/lim AND [english]/lim |

# Table S3. Summary results for all included studies

| **Reference** | **Country** | **Sample** | **Experimental design** | **Group exposure** | **Duration** | **Devices/motion capture** | **measure(s)** | **Outcomes** |
| --- | --- | --- | --- | --- | --- | --- | --- | --- |
| Annerstedt et al., 2013 | Sweden | N=30 (30 males) Mean age: 27.7, SD: 6.7  Healthy students and colleagues | randomized parallel group | EG1: forest visual setting with forest congruent sounds  EG2: only forest visual setting  CG: no forest or forest congruent sounds | 40min | CAVE; Head motion capture | Saliva cortisol, HR, HRV, TWA, STAI-S | - Simulated nature increased para-sympathetic activity and more effectively recovered from virtual stress. |
| Bielinis et al., 2020 | Finland | N=42 (19 males) Mean age: 26.24, SD: 6.23  University students | randomized crossover | EG: Kruunuvuorenranta area with canopy layer of a forest area and Helsinki Central Park  CG: urban environment in East-Helsinki | 15min  Repeated in other setting after at least 24h | Non-surround screen; No motion capture | POMS, SVS | - The values of tension, fatigue, forgetfulness, and irritation significantly decreased after watching the forest video  -No significant difference between settings was observed in the SVS subscale |
| Bornioli et al., 2018 | UK | N=269 (83 males) Mean age: 31.69, SD: 13.63 Age range:18-67  University students (n=129); employees of organizations (n=140) | randomized parallel group | EG1: pedestrianized historic environment EG2: pedestrianized modern environment EG3: pedestrianized mixed environment EG4: an inner city park CG: a commercial area with traffic | 1min | Non-surround screen; No motion capture | UWIST-MACL | - Those who simulated walking in the urban park reserve experienced a more hedonic tone than those in the traffic-filled area  -Relaxation levels did not significantly change in city parks but decreased in the traffic area |
| Brancato et al., 2022 | USA | N=202 (108 males) Mean age: 40.43, SD: 11.66 Age range: 21-72 | randomized parallel group | EG1: pine forest in Oregon  EG2: a farmed field  EG3: the tree-lined Beacon Hill neighborhood of Boston  CG: New York city, including Times Square and midtown Manhattan | 15min | Non-surround screen; No motion capture | VAS, STAI, PANAS | -The pine forest rated more highly concerning happiness than the urban environment  - Sadness ratings did not show a significant main effect of time and condition  - Natural environment increased calmness ratings and decreased anxiety ratings, while in the urban environment, the opposite trend emerged. |
| Browning et al., 2019 | USA | N=89 (44 males) Mean age: 20.2, SD: 1.4 Age range: 18-27  University students | randomized parallel group | EG: Bottomland oakhickory forest  CG: a blank white wall | 6 min | HMD; Head motion capture | EDA, PANAS | -An increase in physiological arousal during exposure to virtual nature was associated to a lesser extent with an increase or maintenance of positive affective states |
| Chan et al., 2021a | Singapore | N=111 (40 males) Mean age: 21.63, SD: 1.81  University students | randomized parallel group | EG: vertical greenery  CG: Corresponding shades of green | 5min | HMD; Head and Body motion capture | HRV, PANAS, STAI | -Positive affect was significantly lower in the control condition but not significantly changed in the vertical greenery condition  -No main effect of the condition was found in negative affect and anxiety |
| Chan et al., 2021b (study 1) | Singapore | N=30 (9 males) Mean age: 20.5, SD: 1.5  University students | randomized crossover | EG: a path between trees in a forest  CG: a path between buildings in a downtown | 5min  Repeated in other setting within a week | HMD; Head and Body motion capture | HRV, PANAS | - Positive affect was not significantly changed by the nature condition but decreased following the urban condition  -Simulated nature helped reduce negative emotions and relieve physical stress compared to urban conditions |
| Chan et al., 2021b (study 2) | Singapore | N=20 (2 males) Mean age: 72.7, SD: 8.8  Seniors | randomized crossover |  | 3min  Repeated in other setting within a week | HMD; Head motion capture | HRV, Simplified scales (positive affect, self‑reported stress) | -Participants rated higher on the positive affect in the nature condition compared to the urban condition  - Stress decreased in nature and increased in urban areas, but both did not reach conventional significance |
| Jiang et al., 2021 | USA, Hong Kong | N=137 (48 males)  University students | randomized parallel group | 3 (visual settings) $\times$ 4 (auditory stimuli)  Visual settings: urban park, office plaza, urban street Auditory stimuli: mute, mechanical sound, traffic sound, nature sound | 5min | Non-surround screen; No motion capture | MDMQ | -Natural audio-visual content increased positive mood, while human-related audio-visual content increased negative mood |
| Jo et al., 2022 | Korea | N=60 (30 males) Mean age: 24.3, SD: 2.4  University students with mild depression, stress, and anxiety | randomized crossover | EG1: green spaces  EG2: waterfront spaces  CG: high- and low-density commercial and business areas | 3min  Repeated 3 times, provided breaks as needed | HMD; Head motion capture | K-POMS, HRV | -The natural environment created in the audio-visual environment can reduce negative emotional responses, induce a physiological and psychological recovery effect |
| Kimura et al., 2021 | Japan | N=30 (15 males) Age range: 20-29  Undergraduate and graduate students | randomized crossover | EG: forest scenes CG: a gray background | 5min  Repeated in other setting after a 3h break | Non-surround screen; No motion capture | EDA, Arousal and valence Subjective Rating | -Viewing nature videos did not affect subjective mental workload but reduced SCL |
| Kinnafick and Thøgersen-Ntoumani, 2014 | UK | N=40 (8 males) Mean age: 23, SD: 7.65 Age range: 18-60  staff and students from the University | randomized crossover | EG: a park primarily made up of green space with small areas of woodland and a pond.  CG: a busy commercially dominant area | 15min  Repeated in other setting after 1 week | Non-surround screen; No motion capture | PANAS, AD ACL, FS, FAS | -Viewing natural scenes significantly reduced negative affect, which increased in urban scenes  -Compared to urban scenes, sitting and watching simulated nature made people calmer and less tension |
| McMahan et al., 2018 | USA | N=89 (31 males) Mean age: 24.22, SD: 7.43  University students | randomized parallel group | EG: five natural environment  CG: five built environment | 6min | HMD; Head motion capture | PANAS | -Type of environment has no major effect on positive and negative affect |
| Meuwese et al., 2021 (study 1) | Netherlands | N=57 (15 males) Mean age: 19.91, SD: 2.76 age range: 18-37  31.60% participants had depressive symptoms | randomized parallel group | EG: a paved road through a forest of the “National Park de Hoge Veluwe”  CG: the city of The Hague | 8min | Non-surround screen; No motion capture | ZIPERS, POMS-SF | -Participants experienced less stress after viewing the walking video in nature compared to the built environment.  -Positive affect was slightly higher in the nature condition but did not reach statistical significance |
| Meuwese et al., 2021 (study 2) | Netherlands | N=200 (109 males) Mean age: 34.94, SD: 11.67 age range: 20-68  26.50% participants had a clinically relevant number of depressive symptoms | randomized parallel group |  | 8min | Non-surround screen; No motion capture | ZIPERS, POMS-SF | Little difference was found between the experienced stress in nature and the built conditions for those with low depressive symptoms. However, those with high depressive symptoms experienced more post-viewing stress in the built condition but not in the natural condition. |
| Mostajeran et al., 2021 | German | N=34 (23 males) Mean age: 27.26, SD: 4.144  University students | non-randomized crossover | 2 (environment type)$\times$2 (presentation formats) with a CG Environment type: Forest, urban environment Presentation format: photo slideshows, 360◦ videos CG: a black room with a white screen in its center | 6min Repeated 4 times | HMD; Head motion capture | EDA, HR, POMS, STADI-S, SSSQ | -Exposure to urban environments was more emotionally disruptive than forests.  -No significant effects of environmental conditions were found in STADI-S, SSSQ, PSS, HR, and SCR. |
| Newman et al., 2022 (study 2) | UK | N=120 (16 males) Mean age: 20, SD: 2.52 | randomized parallel group | 2 (realism) $\times$ 2 (environment type)  Realism: high realism, low realism Environment type: nature, built environment | 10min walking | HMD; Head and Body motion capture | PANAS | -In positive affect, there was a significant main effect of environment type. However, this did not apply to negative affect  -No significant main effect of level of realism was observed |
| O’Meara et al., 2020 | Ireland | N=40 (10 males) Mean age: 20.4, SD: 1.15 Age range:18-24  Half of the participants had high exam anxiety, half had low exam anxiety | randomized parallel group | EG: a lush green forest in Coombaune Woods  CG: an alleyway of an urban town in Killorglin | 4min | HMD; Head motion capture | PANAS | -Virtual natural exposure reduced negative emotions in high anxiety students rather than in low anxiety students  -The nature-based VR intervention did not significantly increase positive affect scores |
| Pilotti et al., 2014 | USA | N=63 (24 males) Mean age: 31.79, SD: 9.42  Student advisors | randomized parallel group | EG: a nature trail  CG: a busy city street | 15min | Non-surround screen; No motion capture | SBP, DBP, PANAS, PAS | - SBP was increased in participants exposed to nature but was kept stable in those exposed to urban environments.  -Self-reported outcomes and physiological indicators outcomes did not appear to be correlated. |
| Schutte et al., 2017 | Australia | N=26 (10 males) Mean age: 34.46, SD: 12.60  University students | randomized parallel group | EG: Australian nature landscape  CG: a small Australian town | 6min | HMD; Head motion capture | PANAS | -The virtual natural environments had a positive influence on positive affect and restoration, but not on negative affect when compared with the virtual urban environments |
| Snell et al., 2018 | Australia | N=60 (22 males) Mean age: 25.43, SD: 9.15 | randomized parallel group | EG1: a video recording natural environment  EG2: the same scene being streamed live from another location  CG: A blank screen | 5min | Non-surround screen; No motion capture | HR, HRV, EDA | -SCL dropped more rapidly in the experimental groups than in the control group  -No significant interaction between settings was  observed in HR and HRV. |
| Sona et al., 2019 | German | N=122 (58 males) Mean age: 22.69, SD: 2.23  Students | randomized parallel group | 2 (environment type) $\times$ 2 (sensory input) with a CG Environment type: park, built indoor environment Sensory input: audio-visual input, audio-visual and olfactory input CG: no audio-visual and olfactory input | 15min | Non-surround screen; No motion capture | Nitsch's Personal State Scale | -A sensory-enriched simulated natural or lounge environment benefits the recovery of personal resources |
| van den Berg et al., 2003 | Netherland | N=106 (34 males) Mean age: 21.9 | randomized parallel group | EG: a path in the forest area  CG: a nearby street | 7min | Non-surround screen; No motion capture | POMS, self-report overall stress and happiness | -Viewing the natural environment showed greater resilience on all five affective measures than viewing the built environment |
| Van den Berg et al., 2014 | UK | N=102 (48 males) Mean age: 22.2 Age range:17-40  University students | randomized parallel group | EG1: Sheffield Graves Park  EG2: Sheffield Botanical Gardens  EG3: Sheffield Greno Woods  CG: an urban street in the city of Sheffield. | 6min40s | Non-surround screen; No motion capture | POMS-SF | -All three nature conditions enhanced positive affect and vitality more than urban street scenes |
| Wang et al., 2016 | China | N=140 (70 males) Mean age: 22.38, SD: 2.56  University students | randomized parallel group | EG1: lawn without people EG2: lawn with people EG3: plaza without people EG4: plaza with people EG5: small lake without people EG6: walkway without people CG: urban roadway | 8min | Non-surround screen; No motion capture | EDA, HRV, STAI-S | -The urban park scenes significantly reduced subjects' stress, state-anxiety and were highly rated in perceived restorativeness than the urban roadway scene |
| Yu et al., 2018 | Taiwan | N=30 (13 males) The majority of (93.3%) peoples' age range: 20-29  Healthy individuals | randomized crossover | EG: Aowanda National Forest Recreation Area at Nantao, Taiwan CG: urban setting in Ximending at Taipei, Taiwan | 9min30s Repeated in other setting after 1 week | HMD; Head motion capture | HRV, Salivary α amylase, SBP, DBP, POMS | -The forest environment significantly increased positive psychological responses, while the urban environment made participants fatigued and grew angry and hostile  -Participants' confusion, stress, and depression in both settings decreased in the posttest |
| Yu et al., 2020 | Taiwan | N=106 (67 males) at least 45 years old  Healthy individuals | randomized crossover | EG: settings containing natural elements  CG: settings including buildings, signs, crowds, and traffic noises | 10min  Repeated in other setting after 1 week | HMD; Head motion capture | HR, HRV, SBP, DBP, POMS | -No differences were observed in physiological responses between the two scenes.  -Exposure to virtual natural environments was associated with more positive feelings and lower levels of fatigue and depression. |
| Zabini et al., 2020 | Italy | N=75 (31 males) Mean age: 47.3, SD: 13.1 | randomized parallel group | EG: forest environment in the Tuscany region  CG: urban environment in downtown Prato, Tuscany, Italy | 5min6s  exposure once a day for 5 days | Non-surround screen; No motion capture | SPRAS, STAI | -Self-perceived relaxation effects were induced by the forest video but not by the urban video |

EG: Experimental Group; CG: Control Group; CAVE: Cave Automatic Virtual Environment; HMD: Head-Mounted Display; PANAS: Positive and Negative Affect Schedule; POMS: Profile of Mood States; K-POMS: Korean edition of Profile of Mood States; POMS-SF: the short form of the Profile of Mood States; STAI: State-Trait Anxiety Inventory; STAI-S: the state scale of the State-Trait Anxiety Inventory; STADI-S: State-Trait Anxiety Depression Inventory-State; ZIPERS: Zuckerman's inventory of personal reactions scale; SVS: Subjective Vitality Scale; SPRAS: Sheehan Patient Rated Anxiety Scale; PAS: Perceived Arousal Scale; UWIST-MACL: the University of Wales Institute of Science and Technology Mood Adjective Checklist; AD ACL: The Activation-Deactivation Adjective Check List; FS: Feelings Scale; FAS: Felt Arousal Scale; VAS: Visual Analogue Scale; MDMQ: Multi-Dimensional Mood Questionnaire; HR: Heart Rate; HRV: Heart Rate Variability; SBP: Systolic Blood Pressure; DBP: Diastolic Blood Pressure; EDA: Electrodermal Activity; TWA: T-wave amplitude.

**Table S4.** ROBINS-I assessments

| **Reference** | **confounding** | **selection of participants into the study** | | **classification of interventions** | | **deviations from intended intervention** | **missing data** | **measurement of outcomes** | **selection of the reported result** | **Overall**  **bias** |
| --- | --- | --- | --- | --- | --- | --- | --- | --- | --- | --- |
| Mostajeran et al. 2021 | Moderate | Low | Low | | Low | | Low | Low | Moderate | Moderate |

**Table S5.** Interpretation of the main outcomes

| **Main outcomes** | **Interpretation** |
| --- | --- |
| Positive affect | The pleasurable affective states that individuals experience as a result of internal and external stimuli. Positive affect can be interpreted as high order dimensions, including high activated positive affect (e.g., vigorous, excited), medium activated positive affect (e.g., happy, pleased) and low pole of positive affect (e.g., calm). |
| Negative affect | The individual's upset or unpleasant affective states due to internal and external stimuli. It is also a high order dimensions, including high activated negative affect (e.g., tensive, anger), medium activated negative affect (e.g., miserable, unhappy) and low activated negative affect (e.g., depressive). |
| Self-reported stress | A comprehensive psychological state that makes individuals feel tense and threatened, which is reflected through self-report. |
| SBP | The pressure of blood against the inner walls of blood vessels when the heart contracts and the pressure in the arteries is at its highest. Elevated SBP may be associated with psychological stress triggered by stimulation from environmental stressors. |
| DBP | The pressure of blood against the inner walls of blood vessels when the heart is diastolic and the arteries are elastic and retracted. Elevated DBP may be associated with psychological stress triggered by stimulation from environmental stressors. |
| HR | The frequency of heartbeats, which is the number of heart beats per minute. HR can be seen as a physiological indicator of stress. |
| LF power | Absolute power of the low-frequency band (0.04–0.15 Hz). It has been proposed that LF power reflects predominantly sympathetic activity or a combination of sympathetic and parasympathetic activity. Activation and mobilization are controlled primarily by the sympathetic branch, whereas relaxation and restoration are controlled primarily by the parasympathetic branch. |
| HF power | Absolute power of the high-frequency band (0.15–0.4 Hz). HF power is suggested to reflect parasympathetic cardiac control, which is mainly associated with restoration and relaxation. |
| LF/HF | Ratio of LF-to-HF power. A low LF/HF ratio reflects parasympathetic dominance and a low LF/HF ratio reflects parasympathetic dominance. |
| RMSSD | Root mean square of successive RR interval differences. The RMSSD is used to estimate a variety of vagally mediated changes in HRV based on the beat-to-beat variance in HR. A higher RMSSD is associated with improved automatic nervous system function, which increases stress tolerance. |

**Table S6.** Sensitivity analysis of the effects on positive affect

| **Excluded study** | **Effect SMD** | **95% CI** | **Heterogeneity I^2^ (%)** |
| --- | --- | --- | --- |
| None | 0.40 | (0.22, 0.58) | 37 |
| Browning et al. 2019 | 0.34 | (0.18, 0.49) | 11 |
| Chan et al. 2021a | 0.43 | (0.22, 0.64) | 43 |
| Chan et al. 2021b (study1) | 0.42 | (0.21, 0.62) | 43 |
| Chan et al. 2021b (study2) | 0.36 | (0.19, 0.53) | 26 |
| Kinnafick and Thøgersen-Ntoumani 2014 | 0.44 | (0.25, 0.64) | 38 |
| Meuwese et al. 2021 (study1) | 0.43 | (0.23, 0.63) | 42 |
| Meuwese et al. 2021 (study2) | 0.44 | (0.24, 0.65) | 38 |
| Newman et al. 2022 (study2) | 0.44 | (0.25, 0.63) | 38 |
| Newman et al. 2022 (study2) | 0.44 | (0.24, 0.63) | 40 |
| O’Meara et al. 2020 | 0.41 | (0.21, 0.61) | 43 |
| Pilotti et al. 2014 | 0.39 | (0.19, 0.58) | 38 |
| Schutte et al. 2017 | 0.39 | (0.20, 0.57) | 38 |

**Table S7.** Sensitivity analysis of the effects on negative affect

| **Excluded study** | **Effect SMD** | **95% CI** | **Heterogeneity I^2^ (%)** |
| --- | --- | --- | --- |
| None | -0.09 | (-0.23, 0.05) | 0 |
| Browning et al. 2019 | -0.12 | (-0.26, 0.03) | 0 |
| Chan et al. 2021a | -0.11 | (-0.26, 0.04) | 0 |
| Chan et al. 2021b (study1) | -0.09 | (-0.23, 0.05) | 0 |
| Kinnafick and Thøgersen-Ntoumani 2014 | -0.06 | (-0.21, 0.08) | 0 |
| Meuwese et al. 2021 (study1) | -0.09 | (-0.23, 0.05) | 0 |
| Meuwese et al. 2021 (study2) | -0.10 | (-0.26, 0.06) | 0 |
| Newman et al. 2022 (study2) | -0.12 | (-0.27, 0.02) | 0 |
| Newman et al. 2022 (study2) | -0.08 | (-0.22, 0.06) | 0 |
| O’Meara et al. 2020 | -0.07 | (-0.21, 0.07) | 0 |
| Schutte et al. 2017 | -0.08 | (-0.22, 0.06) | 0 |
| Van den Berg et al. 2014 | -0.07 | (-0.22, 0.07) | 0 |

**Table S8.** Sensitivity analysis of the effects on perceived stress

| **Excluded study** | **Effect SMD** | **95% CI** | **Heterogeneity I^2^ (%)** |
| --- | --- | --- | --- |
| None | -0.38 | (-0.71, -0.06) | 0 |
| Chan et al. 2021b (study2) | -0.36 | (-0.74, 0.03) | NA |
| van den Berg et al. 2003 | -0.46 | (-1.09, 0.17) | NA |

**Table S9.** Sensitivity analysis of the effects on total mood disturbance (TMD)

| **Excluded study** | **Effect SMD** | **95% CI** | **Heterogeneity I^2^ (%)** |
| --- | --- | --- | --- |
| None | -0.87 | (-1.17, -0.57) | 0 |
| Jo et al. 2022 | -0.84 | (-1.34, -0.35) | NA |
| Yu et al. 2020 | -0.89 | (-1.26, -0.51) | NA |

**Table S10.** Sensitivity analysis of the effects on tension

| **Excluded study** | **Effect SMD** | **95% CI** | **Heterogeneity I^2^ (%)** |
| --- | --- | --- | --- |
| None | -0.70 | (-0.99, -0.41) | 73 |
| Bielinis et al. 2020 | -0.69 | (-1.02, -0.36) | 76 |
| Jo et al. 2022 | -0.65 | (-0.95, -0.34) | 70 |
| Kinnafick and Thøgersen-Ntoumani 2014 | -0.61 | (-0.87, -0.35) | 61 |
| Meuwese et al. 2021 (study1) | -0.77 | (-1.08, -0.45) | 75 |
| Meuwese et al. 2021 (study2) | -0.79 | (-1.08, -0.49) | 65 |
| van den Berg et al. 2003 | -0.75 | (-1.09, -0.41) | 76 |
| Yu et al. 2018 | -0.69 | (-1.02, -0.37) | 76 |
| Yu et al. 2020 | -0.74 | (-1.07, -0.41) | 76 |

**Table S11.** Sensitivity analysis of the effects on fatigue

| **Excluded study** | **Effect SMD** | **95% CI** | **Heterogeneity I^2^ (%)** |
| --- | --- | --- | --- |
| None | -0.60 | (-0.91, -0.28) | 55 |
| Bielinis et al. 2020 | -0.65 | (-1.04, -0.26) | 61 |
| Jo et al. 2022 | -0.55 | (-0.97, -0.13) | 64 |
| Sona et al. 2019 | -0.71 | (-0.94, -0.48) | 5 |
| Yu et al. 2018 | -0.52 | (-0.87, -0.17) | 57 |
| Yu et al. 2020 | -0.53 | (-0.90, -0.16) | 61 |

**Table S12.** Sensitivity analysis of the effects on vigor

| **Excluded study** | **Effect SMD** | **95% CI** | **Heterogeneity I^2^ (%)** |
| --- | --- | --- | --- |
| None | 0.58 | (0.30, 0.86) | 52 |
| Bielinis et al. 2020 | 0.62 | (0.22, 1.01) | 66 |
| Jo et al. 2022 | 0.69 | (0.48, 0.90) | 0 |
| van den Berg et al. 2014 | 0.60 | (0.25, 0.96) | 66 |
| Yu et al. 2018 | 0.53 | (0.22, 0.83) | 56 |
| Yu et al. 2020 | 0.51 | (0.22, 0.81) | 52 |

**Table S13**. Sensitivity analysis of the effects on anxiety

| **Excluded study** | **Effect SMD** | **95% CI** | **Heterogeneity I^2^ (%)** |
| --- | --- | --- | --- |
| None | -0.72 | (-1.43, -0.02) | 91 |
| Brancato et al. 2022 | -0.91 | (-1.96, -0.14) | 93 |
| Chan et al. 2021b | -0.94 | (-1.94, -0.07) | 93 |
| Wang et al. 2016 | -0.28 | (-0.51, -0.06) | 0 |
| Zabini et al. 2020 | -0.83 | (-1.81, 0.16) | 94 |

**Table S14**. Sensitivity analysis of the effects on depression

| **Excluded study** | **Effect SMD** | **95% CI** | **Heterogeneity I^2^ (%)** |
| --- | --- | --- | --- |
| None | -0.33 | (-0.52, -0.14) | 0 |
| Bielinis et al. 2020 | -0.35 | (-0.56, -0.14) | 0 |
| Jo et al. 2022 | -0.35 | (-0.57, -0.13) | 0 |
| van den Berg et al. 2003 | -0.29 | (-0.50, -0.07) | 0 |
| Yu et al. 2018 | -0.36 | (-0.57, -0.16) | 0 |
| Yu et al. 2020 | -0.31 | (-0.52, -0.11) | 0 |

**Table S15**. Sensitivity analysis of the effects on confusion

| **Excluded study** | **Effect SMD** | **95% CI** | **Heterogeneity I^2^ (%)** |
| --- | --- | --- | --- |
| None | -0.79 | (-1.19, -0.40) | 54 |
| Jo et al. 2022 | -0.59 | (-0.95, -0.24) | 0 |
| Yu et al. 2018 | -0.92 | (-1.39, -0.46) | 56 |
| Yu et al. 2020 | -0.85 | (-1.46, -0.24) | 72 |

**Table S16**. Sensitivity analysis of the effects on anger

| **Excluded study** | **Effect SMD** | **95% CI** | **Heterogeneity I^2^ (%)** |
| --- | --- | --- | --- |
| None | -0.54 | (-0.76, -0.31) | 0 |
| Bielinis et al. 2020 | -0.47 | (-0.73, -0.21) | 0 |
| van den Berg et al. 2003 | -0.54 | (-0.81, -0.26) | 0 |
| Yu et al. 2018 | -0.59 | (-0.84, -0.34) | 0 |
| Yu et al. 2020 | -0.57 | (-0.83, -0.32) | 0 |

**Table S17**. Sensitivity analysis of the effects on happiness

| **Excluded study** | **Effect SMD** | **95% CI** | **Heterogeneity I^2^ (%)** |
| --- | --- | --- | --- |
| None | 0.26 | (0.00, 0.51) | 0 |
| Brancato et al. 2022 | 0.30 | (-0.08, 0.68) | NA |
| van den Berg et al. 2003 | 0.22 | (-0.12, 0.56) | NA |

**Table S18**. Sensitivity analysis of the effects on calmness

| **Excluded study** | **Effect SMD** | **95% CI** | **Heterogeneity I^2^ (%)** |
| --- | --- | --- | --- |
| None | 0.54 | (0.17, 0.92) | 63 |
| Brancato et al. 2022 | 0.61 | (0.02, 1.19) | 75 |
| Kinnafick and Thøgersen-Ntoumani 2014 | 0.66 | (0.16, 1.15) | 69 |
| Newman et al. 2022 (study2) | 0.61 | (0.11, 1.12) | 74 |
| Newman et al. 2022 (study2) | 0.37 | (0.13, 0.61) | 0 |

**Table S19**. Sensitivity analysis of the effects on arousal

| **Excluded study** | **Effect SMD** | **95% CI** | **Heterogeneity I^2^ (%)** |
| --- | --- | --- | --- |
| None | -0.10 | (-0.41, 0.21) | 0 |
| Kimura et al. 2021 | -0.02 | (-0.39, 0.35) | 0 |
| Pilotti et al. 2014 | -0.23 | (-0.63, 0.17) | 0 |
| Sona et al. 2019 | -0.06 | (-0.43, 0.31) | 0 |

**Table S20**. Sensitivity analysis of the effects on valence

| **Excluded study** | **Effect SMD** | **95% CI** | **Heterogeneity I^2^ (%)** |
| --- | --- | --- | --- |
| None | 0.40 | (-0.67, 1.48) | 92 |
| Bornioli et al. 2018 | -0.10 | (-0.59, 0.38) | 31 |
| Kimura et al. 2021 | 0.78 | (-0.43, 1.99) | 91 |
| Sona et al. 2019 | 0.53 | (-1.17, 2.22) | 96 |

**Table S21**. Sensitivity analysis of the effects on systolic blood pressure (SBP)

| **Excluded study** | **Effect MD** | **95% CI** | **Heterogeneity I^2^ (%)** |
| --- | --- | --- | --- |
| None | 0.86 | (-3.88, 5.61) | 54 |
| Pilotti et al. 2014 | -0.23 | (-2.04, 1.59) | 0 |
| Yu et al. 2018 | 2.68 | (-4.26, 9.62) | 70 |
| Yu et al. 2020 | 1.85 | (-8.94, 12.64) | 74 |

**Table S22**. Sensitivity analysis of the effects on diastolic blood pressure (DBP)

| **Excluded study** | **Effect MD** | **95% CI** | **Heterogeneity I^2^ (%)** |
| --- | --- | --- | --- |
| None | -0.49 | (-2.71, 1.73) | 0 |
| Pilotti et al. 2014 | -0.13 | (-2.51, 2.24) | 0 |
| Yu et al. 2018 | -0.44 | (-2.93, 2.05) | 0 |
| Yu et al. 2020 | -1.55 | (-5.41, 2.32) | 0 |

**Table S23**. Sensitivity analysis of the effects on heart rate (HR)

| **Excluded study** | **Effect SMD** | **95% CI** | **Heterogeneity I^2^ (%)** |
| --- | --- | --- | --- |
| None | -0.38 | (-0.81, 0.05) | 71 |
| Annerstedt et al. 2013 | -0.50 | (-0.93, 0.08) | 69 |
| Mostajeran et al. 2021 | -0.25 | (-0.70, 0.20) | 67 |
| Mostajeran et al. 2021 | -0.25 | (-0.71, 0.20) | 67 |
| Snell et al. 2018 | -0.44 | (-0.94, 0.05) | 74 |
| Yu et al. 2018 | -0.47 | (-0.95, 0.02) | 71 |
| Yu et al. 2020 | -0.37 | (-0.92, 0.18) | 77 |

**Table S24**. Sensitivity analysis of the effects on low frequency power (LF power)

| **Excluded study** | **Effect SMD** | **95% CI** | **Heterogeneity I^2^ (%)** |
| --- | --- | --- | --- |
| None | 0.22 | (-0.20, 0.64) | 0 |
| Annerstedt et al. 2013 | 0.15 | (-0.33, 0.62) | NA |
| Yu et al. 2020 | 0.46 | (-0.43, 1.35) | NA |

**Table S25**. Sensitivity analysis of the effects on high frequency power (HF power)

| **Excluded study** | **Effect SMD** | **95% CI** | **Heterogeneity I^2^ (%)** |
| --- | --- | --- | --- |
| None | 0.24 | (-0.15, 0.63) | 24 |
| Annerstedt et al. 2013 | 0.12 | (-0.22, 0.47) | 0 |
| Yu et al. 2018 | 0.37 | (-0.49, 1.23) | 62 |
| Yu et al. 2020 | 0.46 | (-0.14, 1.07) | 26 |

**Table S26**. Sensitivity analysis of the effects on low frequency/high frequency (LF/HF)

| **Excluded study** | **Effect SMD** | **95% CI** | **Heterogeneity I^2^ (%)** |
| --- | --- | --- | --- |
| None | -0.10 | (-0.54, 0.34) | 0 |
| Annerstedt et al. 2013 | -0.07 | (-0.58, 0.43) | NA |
| Yu et al. 2018 | -0.18 | (-1.06, 0.70) | NA |

**Table S27**. Sensitivity analysis of the effects on root mean square on successive differences (RMSSD)

| **Excluded study** | **Effect SMD** | **95% CI** | **Heterogeneity I^2^ (%)** |
| --- | --- | --- | --- |
| None | 0.11 | (-0.23, 0.45) | 0 |
| Chan et al. 2021b | -0.14 | (-0.76, 0.49) | NA |
| Snell et al. 2018 | 0.22 | (-0.20, 0.63) | NA |

**Table S28**. Summary of Findings table

| **Certainty assessment** | | | | | | | **№ of patients** | | **Effect** | | **Certainty** | **Importance** |
| --- | --- | --- | --- | --- | --- | --- | --- | --- | --- | --- | --- | --- |
| **№ of studies** | **Study design** | **Risk of bias** | **Inconsistency** | **Indirectness** | **Imprecision** | **Other considerations** | **natural environment** | **non-natural environment** | **Relative (95% CI)** | **Absolute (95% CI)** |  |  |
| **Positive Affect** | | | | | | | | | | | | |
| 11 | randomised trials | very serious^a^ | not serious | not serious | not serious | none | 424 | 425 | - | SMD **0.4 higher** (0.22 higher to 0.58 higher) | ⨁⨁◯◯ Low | CRITICAL |
| **Negative Affect** | | | | | | | | | | | | |
| 10 | randomised trials | very serious^a^ | not serious | not serious | very serious^b^ | none | 450 | 398 | - | SMD **0.09 lower** (0.23 lower to 0.05 higher) | ⨁◯◯◯ Very low | CRITICAL |
| **Self-reported Stress** | | | | | | | | | | | | |
| 2 | randomised trials | very serious^a^ | not serious | not serious | very serious^c^ | none | 73 | 73 | - | SMD **0.38 lower** (0.71 lower to 0.06 lower) | ⨁◯◯◯ Very low | CRITICAL |
| **SBP** | | | | | | | | | | | | |
| 3 | randomised trials | serious^a^ | serious^d^ | not serious | very serious^b^ | none | 95 | 94 | - | MD **0.86 higher** (3.88 lower to 5.61 higher) | ⨁◯◯◯ Very low | IMPORTANT |
| **DBP** | | | | | | | | | | | | |
| 3 | randomised trials | serious^a^ | not serious | not serious | very serious^b^ | none | 95 | 94 | - | MD **0.49 lower** (2.71 lower to 1.73 higher) | ⨁◯◯◯ Very low | IMPORTANT |
| **HR** | | | | | | | | | | | | |
| 4 | randomised trials | serious^a^ | not serious | not serious | very serious^b^ | none | 94 | 94 | - | SMD **0.1 lower** (0.4 lower to 0.2 higher) | ⨁◯◯◯ Very low | IMPORTANT |
| **LF power** | | | | | | | | | | | | |
| 2 | randomised trials | serious^a^ | not serious | not serious | very serious^c^ | none | 44 | 44 | - | SMD **0.22 higher** (0.2 lower to 0.64 higher) | ⨁◯◯◯ Very low | IMPORTANT |
| **HF power** | | | | | | | | | | | | |
| 3 | randomised trials | serious^a^ | not serious | not serious | very serious^b^ | none | 74 | 74 | - | SMD **0.24 higher** (0.15 lower to 0.63 higher) | ⨁◯◯◯ Very low | IMPORTANT |
| **LF/HF** | | | | | | | | | | | | |
| 2 | randomised trials | serious | not serious | not serious | very serious^b^ | none | 40 | 40 | - | SMD **0.1 lower** (0.54 lower to 0.34 higher) | ⨁◯◯◯ Very low | IMPORTANT |
| **RMSSD** | | | | | | | | | | | | |
| 2 | randomised trials | serious | not serious | not serious | very serious^b^ | none | 66 | 65 | - | SMD **0.11 higher** (0.23 lower to 0.45 higher) | ⨁◯◯◯ Very low | IMPORTANT |

**CI:** confidence interval; **MD:** mean difference; **SMD:** standardized mean difference

1. **Explanations**

a. Issues with randomization method, allocation concealment, blinding, incomplete outcome data and selective reporting

b. Credible intervals span clinical decision thresholds for recommended and non-recommended interventions

c. Not meeting the criteria for optimal information content (OIS)

d. The heterogeneity may be substantial and unable to be explained

**Reference**

Annerstedt, M., Jonsson, P., Wallergard, M., Johansson, G., Karlson, B., Grahn, P., et al. (2013). Inducing physiological stress recovery with sounds of nature in a virtual reality forest--results from a pilot study. *Physiol Behav* 118**,** 240-250. doi: 10.1016/j.physbeh.2013.05.023.

Bielinis, E., Simkin, J., Puttonen, P., and Tyrvainen, L. (2020). Effect of Viewing Video Representation of the Urban Environment and Forest Environment on Mood and Level of Procrastination. *Int J Environ Res Public Health* 17(14). doi: 10.3390/ijerph17145109.

Bornioli, A., Parkhurst, G., and Morgan, P.L. (2018). Psychological Wellbeing Benefits of Simulated Exposure to Five Urban Settings: an Experimental Study From the Pedestrian's Perspective. *Journal of Transport & Health* 9**,** 105-116. doi: 10.1016/j.jth.2018.02.003.

Brancato, G., Van Hedger, K., Berman, M.G., and Van Hedger, S.C. (2022). Simulated nature walks improve psychological well-being along a natural to urban continuum. *Journal of Environmental Psychology* 81. doi: 10.1016/j.jenvp.2022.101779.

Browning, M., Mimnaugh, K.J., van Riper, C.J., Laurent, H.K., and LaValle, S.M. (2019). Can Simulated Nature Support Mental Health? Comparing Short, Single-Doses of 360-Degree Nature Videos in Virtual Reality With the Outdoors. *Front Psychol* 10**,** 2667. doi: 10.3389/fpsyg.2019.02667.

Chan, S.H.M., Qiu, L., Esposito, G., and Mai, K.P. (2021a). Vertical greenery buffers against stress: Evidence from psychophysiological responses in virtual reality. *Landscape and Urban Planning* 213. doi: 10.1016/j.landurbplan.2021.104127.

Chan, S.H.M., Qiu, L., Esposito, G., Mai, K.P., Tam, K.P., and Cui, J. (2021b). Nature in virtual reality improves mood and reduces stress: evidence from young adults and senior citizens. *Virtual Real***,** 1-16. doi: 10.1007/s10055-021-00604-4.

Jiang, B., Xu, W., Ji, W., Kim, G., Pryor, M., and Sullivan, W.C. (2021). Impacts of nature and built acoustic-visual environments on human’s multidimensional mood states: A cross-continent experiment. *Journal of Environmental Psychology* 77. doi: 10.1016/j.jenvp.2021.101659.

Jo, H.I., Lee, K., and Jeon, J.Y. (2022). Effect of noise sensitivity on psychophysiological response through monoscopic 360 video and stereoscopic sound environment experience: a randomized control trial. *Sci Rep* 12(1)**,** 4535. doi: 10.1038/s41598-022-08374-y.

Kimura, T., Yamada, T., Hirokawa, Y., and Shinohara, K. (2021). Brief and Indirect Exposure to Natural Environment Restores the Directed Attention for the Task. *Front Psychol* 12**,** 619347. doi: 10.3389/fpsyg.2021.619347.

Kinnafick, F.-E., and Thøgersen-Ntoumani, C. (2014). The effect of the physical environment and levels of activity on affective states. *Journal of Environmental Psychology* 38**,** 241-251. doi: 10.1016/j.jenvp.2014.02.007.

McMahan, E.A., Estes, D., Murfin, J.S., and Bryan, C.M. (2018). Nature connectedness moderates the effect of nature exposure on explicit and implicit measures of emotion. *Journal of Positive Psychology and Wellbeing* 2(2), 128-148.

Meuwese, D., Dijkstra, K., Maas, J., and Koole, S.L. (2021). Beating the blues by viewing Green: Depressive symptoms predict greater restoration from stress and negative affect after viewing a nature video. *Journal of Environmental Psychology* 75. doi: 10.1016/j.jenvp.2021.101594.

Mostajeran, F., Krzikawski, J., Steinicke, F., and Kuhn, S. (2021). Effects of exposure to immersive videos and photo slideshows of forest and urban environments. *Sci Rep* 11(1)**,** 3994. doi: 10.1038/s41598-021-83277-y.

Newman, M., Gatersleben, B., Wyles, K.J., and Ratcliffe, E. (2022). The use of virtual reality in environment experiences and the importance of realism. *Journal of Environmental Psychology* 79. doi: 10.1016/j.jenvp.2021.101733.

O’Meara, A., Cassarino, M., Bolger, A., and Setti, A. (2020). Virtual Reality Nature Exposure and Test Anxiety. *Multimodal Technologies and Interaction* 4(4). doi: 10.3390/mti4040075.

Pilotti, M., Klein, E., Golem, D., Piepenbrink, E., and Kaplan, K. (2014). Is Viewing a Nature Video After Work Restorative? Effects on Blood Pressure, Task Performance, and Long-Term Memory. *Environment and Behavior* 47(9)**,** 947-969. doi: 10.1177/0013916514533187.

Schutte, N.S., Bhullar, N., Stilinović, E.J., and Richardson, K. (2017). The Impact of Virtual Environments on Restorativeness and Affect. *Ecopsychology* 9(1)**,** 1-7. doi: 10.1089/eco.2016.0042.

Snell, T.L., McLean, L.A., McAsey, F., Zhang, M., and Maggs, D. (2018). Nature Streaming: Contrasting the Effectiveness of Perceived Live and Recorded Videos of Nature for Restoration. *Environment and Behavior* 51(9-10)**,** 1082-1105. doi: 10.1177/0013916518787318.

Sona, B., Dietl, E., and Steidle, A. (2019). Recovery in sensory-enriched break environments: integrating vision, sound and scent into simulated indoor and outdoor environments. *Ergonomics* 62(4)**,** 521-536. doi: 10.1080/00140139.2018.1491643.

Van den Berg, A.E., Jorgensen, A., and Wilson, E.R. (2014). Evaluating restoration in urban green spaces: Does setting type make a difference? *Landscape and Urban Planning* 127**,** 173-181. doi: 10.1016/j.landurbplan.2014.04.012.

van den Berg, A.E., Koole, S.L., and van der Wulp, N.Y. (2003). Environmental preference and restoration: (How) are they related? *Journal of Environmental Psychology* 23(2)**,** 135-146. doi: 10.1016/s0272-4944(02)00111-1.

Wang, X., Rodiek, S., Wu, C., Chen, Y., and Li, Y. (2016). Stress recovery and restorative effects of viewing different urban park scenes in Shanghai, China. *Urban Forestry & Urban Greening* 15**,** 112-122. doi: 10.1016/j.ufug.2015.12.003.

Yu, C.-P., Lee, H.-Y., Lu, W.-H., Huang, Y.-C., and Browning, M.H.E.M. (2020). Restorative effects of virtual natural settings on middle-aged and elderly adults. *Urban Forestry & Urban Greening* 56. doi: 10.1016/j.ufug.2020.126863.

Yu, C.-P., Lee, H.-Y., and Luo, X.-Y. (2018). The effect of virtual reality forest and urban environments on physiological and psychological responses. *Urban Forestry & Urban Greening* 35**,** 106-114. doi: 10.1016/j.ufug.2018.08.013.

Zabini, F., Albanese, L., Becheri, F.R., Gavazzi, G., Giganti, F., Giovanelli, F., et al. (2020). Comparative Study of the Restorative Effects of Forest and Urban Videos during COVID-19 Lockdown: Intrinsic and Benchmark Values. *Int J Environ Res Public Health* 17(21). doi: 10.3390/ijerph17218011.
